# FOXA and master transcription factors recruit Mediator and Cohesin to the core transcriptional regulatory circuitry of cancer cells

**DOI:** 10.1038/srep34962

**Published:** 2016-10-14

**Authors:** Michèle Fournier, Gaëlle Bourriquen, Fabien C. Lamaze, Maxime C. Côté, Éric Fournier, Charles Joly-Beauparlant, Vicky Caron, Stéphane Gobeil, Arnaud Droit, Steve Bilodeau

**Affiliations:** 1 Centre de Recherche sur le Cancer de l’Université Laval, Québec, Canada; 2 Centre de recherche du CHU de Québec – Université Laval, Québec, Canada; 3 Département de médecine moléculaire, Faculté de Médecine, Université Laval, Québec, Canada; 4 Département de biologie moléculaire, biochimie médicale et pathologie, Faculté de Médecine, Université Laval, Québec, Canada

## Abstract

Controlling the transcriptional program is essential to maintain the identity and the biological functions of a cell. The Mediator and Cohesin complexes have been established as central cofactors controlling the transcriptional program in normal cells. However, the distribution, recruitment and importance of these complexes in cancer cells have not been fully investigated. Here we show that FOXA and master transcription factors are part of the core transcriptional regulatory circuitry of cancer cells and are essential to recruit M ediator and Cohesin. Indeed, Mediator and Cohesin occupied the enhancer and promoter regions of actively transcribed genes and maintained the proliferation and colony forming potential. Through integration of publically available ChIP-Seq datasets, we predicted the core transcriptional regulatory circuitry of each cancer cell. Unexpectedly, for all cells investigated, the pioneer transcription factors FOXA1 and/or FOXA2 were identified in addition to cell-specific master transcription factors. Loss of both types of transcription factors phenocopied the loss of Mediator and Cohesin. Lastly, the master and pioneer transcription factors were essential to recruit Mediator and Cohesin to regulatory regions of actively transcribed genes. Our study proposes that maintenance of the cancer cell state is dependent on recruitment of Mediator and Cohesin through FOXA and master transcription factors.

Transcription factors enforce the cell-specific transcriptional program by binding regulatory elements dispersed throughout the genome^1^. Indeed, their unique ability to recognize specific DNA sequences makes them the central piece of cell state control. While hundreds of transcription factors are expressed in a cell, only a handful are essential to establish and maintain the transcriptional program^2^,^3^,^4^. A landmark demonstration established decades ago that expression of the transcription factor MYOD1 was alone sufficient to turn fibroblasts into myoblasts^5^. Since then, many transcription factors have been added to the list of the reprogramming transcription factors including the various combinations of OCT4, SOX2, KLF4 and MYC to force somatic cells into an induced pluripotent cell state^6^,^7^. Those dominant transcription factors enforcing a specific transcriptional program are typically part of the core transcriptional regulatory circuitry of a cell^8^. These core transcriptional regulatory circuitries include master transcription factors, like MYOD1 and OCT4, which are expressed at high levels relative to other transcription factors, are essential to cell state maintenance and positively regulate cell-type-specific genes in addition to their own expression^8^,^9^,^10^. Another class of transcription factors important for embryonic development and reprogramming are pioneer transcription factors^11^. These transcription factors, like FOXA1, possess the ability to bind DNA sequences wrapped around nucleosomes and positively or negatively influence the transcriptional program^12^,^13^,^14^. Overall, the intricate interplays between transcription factors define the transcriptional program and, therefore, the cellular state.

Transcription factors exert their control on gene expression through recruitment of a wide variety of cofactors, chromatin modifiers and regulators of the chromosome structure^1^,^8^,^15^. Ultimately, transcription factors are important to recruit the RNA Polymerase II (Pol II) machinery and to regulate elongation^16^,^17^. Bridging the transcription factors with Pol II is the role of the coactivator complex Mediator^18^. Indeed, Mediator serves as a central scaffold in the pre-initiation complex to regulate Pol II activity. Along with the Mediator complex, the Cohesin complex, which is essential to maintain sister chromatids cohesion during cell division^19^, and the cohesin loading factor NIPBL are essential to form cell-type-specific connections between enhancers and promoters^20^. Other transcription factors, like MYC, promotes Pol II pause-release by recruiting the P-TEFb complex at active promoters^21^. Therefore, a combination of transcription factors is required at enhancer and promoter regions to specify the transcriptional program of a cell. Identifying the combinations of transcription factors governing the transcriptional program of each cell provides the core information to control cell state.

The gene expression program controls normal and aberrant biological functions of a cell. Indeed, to reach a disease state, cells often modify their transcriptional program to acquire new identities and functions^8^. Interestingly, modulation of regulatory regions is emerging as a key feature of many human diseases^22^,^23^. For example, the vast majority of single nucleotide polymorphisms (SNPs) associated with cancer are found in the non-coding fraction of the genome with a large subset inside enhancer regions^24^. Furthermore, modulation of enhancer regions is a promising cancer treatment^25^,^26^. Additionally, mutations in transcriptional regulators occupying enhancer regions, including transcription factors and cofactors, are causative of many diseases^27^. Among them, mutations in Mediator, Cohesin and the Cohesin loader NIPBL have been associated to developmental syndromes as well as different forms of cancer^28^,^29^. While it is not clear how these mutations lead to a disease state, they point to the importance of proper control of regulatory regions to maintain a normal cell state.

Here we show that FOXA pioneer transcription factors are, with master transcription factors, the core transcriptional regulatory circuitry of cancer cells. These transcription factors are essential to recruit the Mediator and Cohesin complexes to regulatory regions of actively transcribed genes. Taken together, our results suggest that Mediator and Cohesin are connected to the core transcriptional regulatory circuitry through FOXA and master transcription factors to maintain the cancer cell state.

## Results

### Mediator and Cohesin occupy regulatory regions of actively transcribed genes in cancer cells

**Figure 1:**
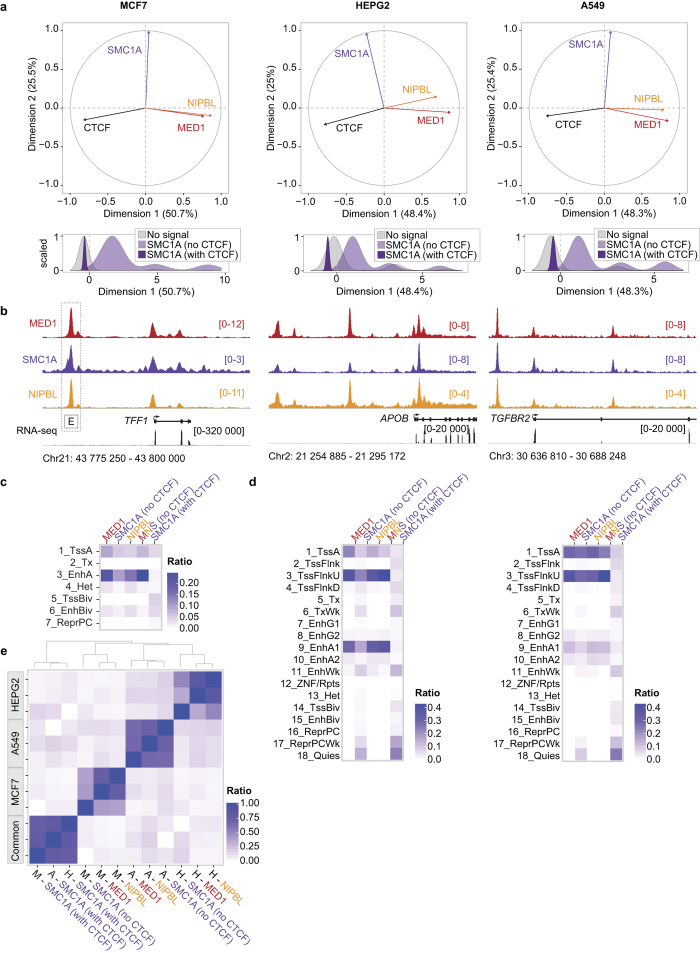
Mediator, Cohesin and NIPBL co-localize at regulatory regions of actively transcribed genes in cancer cells. **(a)** Principal component analysis (PCA) of the distribution of MED1-, SMC1A-, NIPBL- and CTCF-occupied regions. Top – PCA variable factor map of the MED1, SMC1A, NIPBL and CTCF variables on the first two components showing a positive correlation between NIPBL and MED1. In addition, dimension 1 captures the negative correlation between the transcription factor CTCF and MED1/NIPBL-occupied regions. Bottom – Density plot of the genomic region coordinates on the dimension 1 showing a split distribution of SMC1A. SMC1A is associated with CTCF (negative values) or MED1 and NIPBL (positive values). The y axes are normalized to 1 while the x axes represent arbitrary units. **(b)** Occupancy profiles of Mediator (MED1), Cohesin (SMC1A) and NIPBL showing colocalization at regulatory regions. The *TFF1, APOP* and *TGFBR2* loci are displayed for MCF7, HEPG2 and A549 respectively. The characterized enhancer region of *TFF1* (box labeled E) is indicated^60^. The scales of ChIP-Seq and RNA-Seq profiles are displayed in reads per million. **(c)** Overlaps between MED1, SMC1A (no CTCF), SMC1A (with CTCF), the regions co-occupied by MED1-SMC1A-NIPBL and the functional annotation of the MCF7 genome (see Supplementary Information). MED1, SMC1A (no CTCF) and NIPBL occupy active TSS (1_TssA) and active enhancers (3_EnhA), but not inactive regions. The color scale indicates the ratio of overlap. **(d)** Overlaps between MED1, SMC1A (no CTCF), SMC1A (with CTCF), the regions co-occupied by MED1-SMC1A-NIPBL and the functional annotation of the HEPG2 (left) and A549 (right) genomes using the ChromHMM 18-state model^32^,^33^. MED1, SMC1A (no CTCF) and NIPBL occupy active promoters (1_TssA and 3_TssFlnkU) and active enhancer regions (8_EnhG2, 9_EnhA1 and 10_Enh_A2), but not inactive regions in both HEPG2 and A549 cells (see Supplementary Information for abbreviations). The color scales indicate the ratio of overlap. **(e)** Heat map showing the colocalization frequencies of MED1, SMC1A and NIPBL enriched regions in MCF7, HEPG2 and A549 cells. The color scale reflects the colocalization of each pair of regulators. Regulators were clustered along both axes based on the similarity in their correlation with each other.

Maintenance of the transcriptional program depends on transcription factors and cofactors including Mediator and Cohesin. Indeed, Mediator and Cohesin occupy enhancer regions with master transcription factors to control the transcriptional program and identity of embryonic stem cells^20^. To define the contribution of Mediator and Cohesin to the regulation of the cancer transcriptional program, we established the genome-wide occupancy of MED1 (Mediator), SMC1A (Cohesin) and the cohesin loader NIPBL. Three cancer cell lines were profiled using chromatin immunoprecipitation coupled with massively parallel DNA sequencing (ChIP-Seq): MCF7 (breast), HEPG2 (liver) and A549 (lung). Principal component analyses (PCA) confirmed that the distribution profiles of NIPBL and MED1 were positively correlated (R = 0.53, 0.45 and 0.53 for MCF7, HEPG2 and A549, respectively, all p-values < 10e–100) across the genome of each cell type (Fig. 1a) in accordance with previous reports^20^. Furthermore, Cohesin, which is known to occupy regions with NIPBL and Mediator, but also the transcription factor CTCF^30^,^31^, showed a bimodal distribution in all three cell lines (Fig. 1a). Therefore, for subsequent analyses, SMC1A-occupied regions were divided into CTCF-occupied (with CTCF) and not (no CTCF). Detailed examination of the ChIP-Seq density profiles confirmed the presence of Mediator, Cohesin and NIPBL at the transcription start site (TSS) and well-characterized enhancers regions of genes associated with breast, liver and lung functions (Fig. 1b). These results suggest that similar to observations made in primary cells^20^, Mediator and Cohesin co-occupy the genome at regulatory regions of actively transcribed genes in cancer cells.

The mammalian genome is segmented into functional elements orchestrating transcriptional events and gene expression^32^. To extend the occupancy profiles of Mediator, Cohesin and NIPBL genome-wide, we compared the ChIP-Seq data with predicted functional elements. For MCF7 cells, histone marks were combined (**see** Supplementary Information) to define the different chromatin states while an 18-state model built using ChromHMM was available for A549 and HEPG2^32^,^33^. MED1, SMC1A and NIPBL occupied active enhancers and promoters in all three cell types (Fig. 1c,d). Lowly transcribed regions including heterochromatin and polycomb-occupied sites were not enriched. While the functional elements occupied by Mediator, Cohesin and NIPBL are similar in the three cancer cells investigated, the coordinates of these elements are different. Indeed, regions occupied by MED1, SMC1A (no CTCF) and NIPBL in MCF7 were different from those found in HEPG2 and A549 (Fig. 1e). However, regions occupied by CTCF and SMC1A (SMC1A with CTCF) were highly redundant between cell lines in accordance with previous reports^34^. Taken together, these results suggest a conserved function of Mediator, Cohesin and NIPBL in the control of regulatory elements throughout the genome of cancer cells.

### Proliferation of cancer cells depends on Mediator and Cohesin

**Figure 2:**
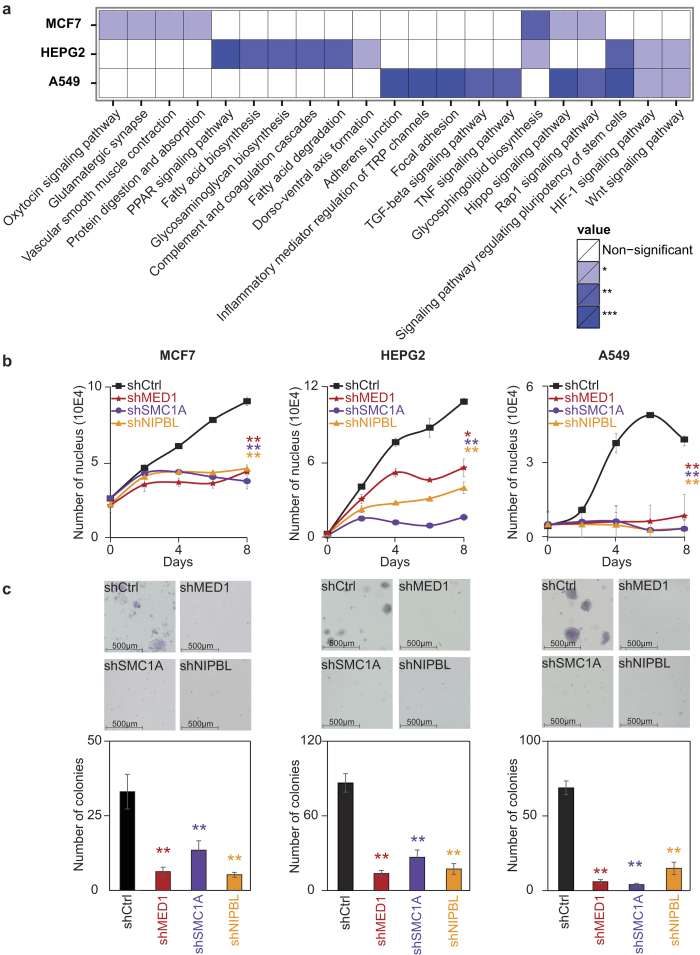
Mediator, Cohesin and NIPBL are essential for maintenance of cancer cells properties. **(a)** Heat map representation of KEGG pathways enriched for genes occupied by MED1, SMC1A and NIPBL in MCF7, HEPG2 and A549 cells (Fisher’s exact test, *p < 0.05, **p < 0.01, ***p < 0.001). **(b)** Growth curve of MCF7, HEPG2 and A549 following decreased expression of MED1, SMC1A and NIPBL showing a global negative effect on cell growth. Each line represents the average of a minimum of two biological replicates for two independent shRNA constructs. The p-values were calculated by comparing with the shRNA controls at day 8 (paired t-test, *p < 0.05 and **p < 0.01). **(c)** Soft agar colony formation assays with decreased levels of MED1, SMC1A and NIPBL showing a loss of colony formation potential. Colonies were quantified with the ImageJ software using 4 images per well. The bar graph represents the average of a minimum of two biological replicates for two independent shRNA constructs. The p-values were calculated by comparing with the shRNA controls (paired t-test, *p < 0.05 and **p < 0.01).

As in normal cells, the gene networks controlling the identity and functions of each cancer cell is unique. To determine the nature of the genes occupied by Mediator, Cohesin and NIPBL, we investigated the associated gene networks in MCF7, HEPG2 and A549. KEGG pathways analyses revealed the association of MED1, SMC1A and NIPBL with genes reflecting the normal and disease functions of the tissues (Fig. 2a and Supplementary Table S1). For example, in MCF7 (breast) cells, genes implicated in the oxytocin signaling pathway were enriched. For HEPG2 (liver) cells, genes associated with hepatic functions like PPAR signaling, complement and coagulation cascades as well as fatty acids metabolism were strongly enriched. In A549 (lung) cells, pathways associated with cell adhesion and TGF-beta signaling were identified. Interestingly, among the direct target genes, many known oncogenes including FGFR2, MAX and PIK3R1 in MCF7, PDGFB, PPARG and HIF1A in HEPG2 and MYC, MET and EGFR in A549 were identified. These observations suggest a role of Mediator and Cohesin in the maintenance of cancer cell properties through a cell-specific network of genes.

Whether or not cancer cells are dependent on Mediator, Cohesin and NIPBL to maintain their proliferative state is not known. Therefore, we quantified the cellular response of MCF7, HEPG2 and A549 with proliferation and soft-agar colony formation assays in MED1, SMC1A and NIPBL loss-of-function experiments. For each target, two independent shRNAs were validated for efficiency by Western blot and used in functional assays (Supplementary Fig. S1). In all three cell lines, similar results were obtained (Fig. 2b,c). Indeed, loss of MED1, SMC1A or NIPBL led to a global decrease in proliferation (Fig. 2b). During the 7-day experiment, cells were not dividing, but were maintained. To determine if these changes affected the colony formation potential of cancer cells, we used soft-agar colony formation assays. Loss of MED1, SMC1A and NIPBL reduced the colony formation potential of MCF7, HEPG2 and A549 (Fig. 2c). Indeed, all cells transduced with control shRNAs produced colonies while their number were significantly reduced with decreasing levels of MED1, SMC1A and NIPBL. Altogether, these results support a role of Mediator and Cohesin in the proliferation of cancer cells.

### Mediator and Cohesin connect with the core transcriptional regulatory circuitry of cancer cells

**Figure 3:**
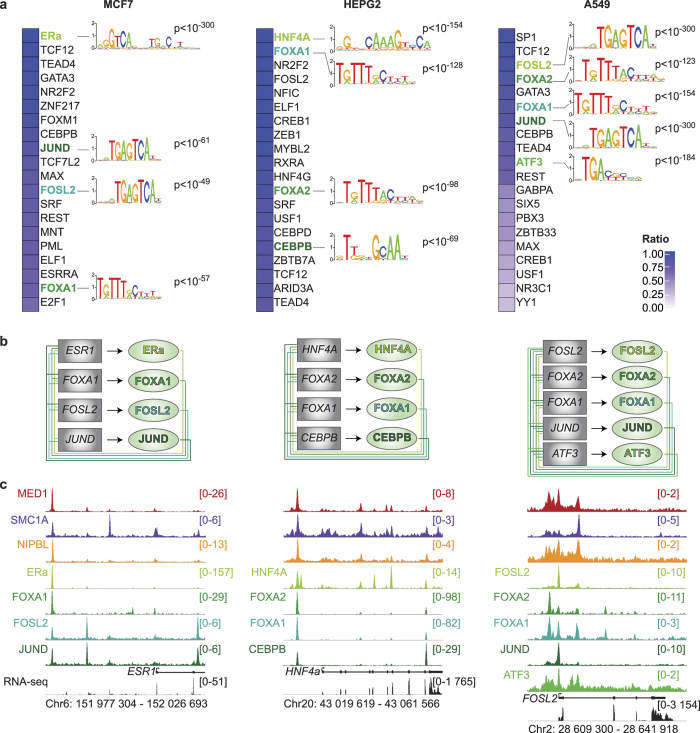
The core circuitry of cancer cells connect with Mediator and Cohesin. **(a)** Percentage of overlapping regions occupied by MED1, SMC1A and NIPBL with transcription factors. The best 20 overlaps with transcription factors for each cell type are presented. The color scale indicates the percentage of overlap. The DNA binding motif logos found in the regions occupied by MED1, SMC1A and NIPBL are indicated next to their associated transcription factor. The motifs were found in the top 20 of the most enriched position-weight matrices. The associated p-values are indicated. **(b)** Core transcriptional regulatory circuitry of MCF7, HEPG2 and A549 cells. The transcription factors identified in (**a**) are interconnected. The box represents the transcription factor gene loci while the oval represents the protein identified in (**a**). Each line represents the interaction of a transcription factor with the indicated gene loci (−10 kb to the end of the gene). **(c)** Occupancy profiles of Mediator (MED1), Cohesin (SMC1A) and NIPBL in addition to the core transcription factors identified in (**a**) showing colocalization. The *ESR1, HNF4A* and *FOSL2* loci are displayed for MCF7, HEPG2 and A549 respectively. The scales of ChIP-Seq profiles are displayed in reads per million.

Cofactors are recruited to regulatory elements by transcription factors. To determine the transcription factor(s) recruiting Mediator and Cohesin, we collected publicly available ChIP-Seq datasets for transcription factors (Supplementary Table S2) and computed pairwise genome-wide relationships for each cell line. Transcription factors were ranked based on their percentage of overlap with MED1, SMC1A and NIPBL across the genome of each cancer cell (Fig. 3a). The top 20 was selected for further filtering. Transcription factors are recruited to functional elements in *cis* through recognition of a DNA sequence but also in *trans* through protein-protein interactions^35^. To refine the list of candidates, we performed an *in silico* enrichment of the binding motifs found in the HOCOMOCO database in regions occupied by MED1, SMC1A and NIPBL. The 20 most frequent DNA motifs found in regions occupied by MED1, SMC1A and NIPBL were cross-referenced with the list of 20 co-occupying transcription factors (Fig. 3a). In each cell line, multiple transcription factors were identified. In MCF7, ERa, FOXA1, FOSL2 and JUND were found, while HNF4A, FOXA1, FOXA2 and CEBPB were identified in HEPG2 and FOSL2, FOXA1, FOXA2, JUND and ATF3 in A549. These results suggest that a combination of transcription factors is responsible for the recruitment of Mediator and Cohesin at regulatory regions.

Whether the identified transcription factors formed a core transcriptional regulatory circuitry in each cancer cell is unknown. To determine the connections between the transcription factors and their corresponding genes, we built the transcriptional network using the ChIP-Seq data for each transcription factor (Fig. 3b and Supplementary Fig. S2). Interestingly, the transcription factors were interconnected in all three cell types (Fig. 3b). In MCF7, ERa was found at the promoter of *ESR1, FOSL2, FOXA1* and *JUND*. In return, FOSL2, FOXA1 and JUND occupied the promoter of *ESR1* (Fig. 3c and Supplementary Fig. S2). The connections for MCF7, HEPG2 and A549 are illustrated in Fig. 3b,c. These results suggest that the transcription factors underlying the recruitment of Mediator and Cohesin are part of the core transcriptional regulatory circuitry of cancer cells.

### FOXA and master transcription factors are essential to cancer cell maintenance

To prioritize candidates for functional studies, we wanted to identify the central and most important transcription factors in each network. Interestingly, many of the identified transcription factors are or are presumed to be master and pioneer transcription factors. For example, ERa and HNF4A were implicated with the core transcriptional regulatory circuitry of breast and liver cells^14^,^36^. A shared characteristic of master transcription factors is that they form positive autoregulatory loops, regulating their own gene^2^. Therefore, we identified the transcription factors occupying their own regulatory regions with Mediator, Cohesin and NIPBL. A limited number of transcription factors fulfilled that characteristic and they became our leading candidates for controlling cancer cells. These transcription factors included ERa in MCF7; HNF4A and FOXA2 in HEPG2 and FOSL2 in A549 (Fig. 3b,c). Since, the FOXA family of pioneer transcription factors was identified in all cell lines, FOXA1 in MCF7 and FOXA2 in A549 were also included for further characterization. It is to be noted that many of these transcription factors are associated with Mediator and Cohesin in various cell types. Indeed, ERa and HNF4A were shown to interact with Mediator^37^,^38^,^39^. In addition, ERa was co-purified with Cohesin subunits^40^. Furthermore, FOXA1 and FOXA2 were found to recruit coactivators and interact with other transcription factors at functional enhancers^35^,^41^,^42^.

**Figure 4:**
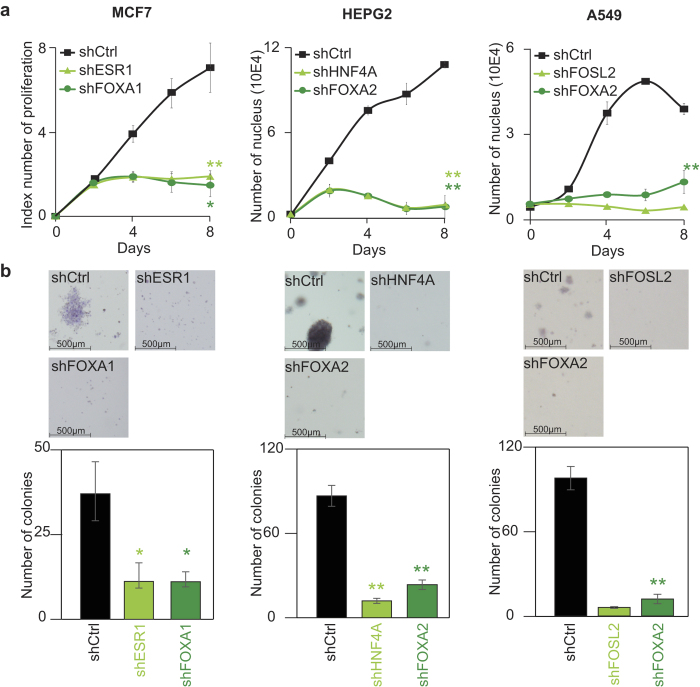
Depletion of the core transcription factors phenocopies the loss of Mediator and Cohesin. **(a)** Growth curve of MCF7, HEPG2 and A549 following decreased expression of ERa and FOXA1 in MCF7, HNF4A and FOXA2 in HEPG2 and FOLS2 and FOXA2 in A549. The results show a global negative effect on cell growth. Each line represents the average of a minimum of two biological replicates for two independent shRNA constructs with the exception of FOSL2 where one shRNA construct was used. The p-values were calculated by comparing with the shRNA controls at day 8 (paired t-test, *p < 0.05 and **p < 0.01). **(b)** Soft agar colony formation assays showing a loss of colony formation potential with decreased expression of core transcription factors. Colonies were quantified with the ImageJ software using 4 images per well. The bar graph represents the average of a minimum of two biological replicates for two independent shRNA constructs with the exception of FOSL2 where one shRNA construct was used. The p-values were calculated by comparing with the shRNA controls (paired t-test, *p < 0.05 and **p < 0.01).

If the identified transcription factors are essential to the function of Mediator, Cohesin and NIPBL, they should phenocopy each other in functional loss-of-function assays. Indeed, we have shown that Mediator and Cohesin are essential to the proliferation of the cancer cells (Fig. 2). Therefore, we used the same cell proliferation and colony formation assays with validated shRNAs targeting each identified transcription factors (Supplementary Fig. S3). In all cases, loss of the transcription factor reduced the proliferation and the colony formation potential of cancer cells (Fig. 4a,b). Indeed, lower levels of ERa and FOXA1 in MCF7, HNF4A and FOXA2 in HEPG2 and FOSL2 and FOXA2 in A549 reduced the proliferation rate (Fig. 4a) and the colony formation potential (Fig. 4b). The results demonstrate that the identified core transcriptional regulatory circuitry in each cell is essential to maintain cancer cells.

### FOXA and master transcription factors recruit Mediator and Cohesin

**Figure 5:**
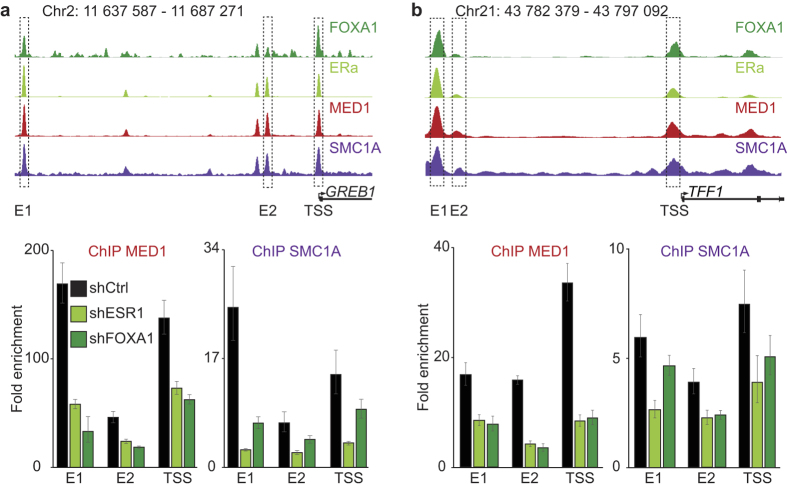
FOXA and master transcription factors are essential to recruit Mediator and Cohesin. Depletion of either the master transcription factor ERa or the pioneer transcription factor FOXA1 in MCF7 cells decreases MED1 and SMC1 recruitment at regulatory elements of the *TFF1* and *GREB1* genes in MCF7. Amplified enhancer (E1 and E2) and TSS regions are indicated by boxes. Enrichment fold were calculated relative to control regions without MED1 or SMC1 signal. The bar graphs display the enrichment fold of a representative experiment. Error bars show the standard error of the mean for technical qPCR triplicates.

Integration of the ChIP-Seq results and the functional experiments suggest that Mediator and Cohesin are recruited to regulatory regions of actively transcribed genes by the core transcriptional regulatory circuitry. To functionally validate our results, we focused our analysis on MCF7 cells. Pioneer transcription factors like FOXA1 are known to bind the condensed chromatin prior to transcriptional activation^12^. On the other hand, master transcription factors like ERa tend to occupy nucleosome-free regions^12^. We entertained two models: (1) an all-or-nothing model where the presence of many transcription factors is required to stabilize large cofactors complexes like Mediator and Cohesin and (2) a sequential model where the transcription factors and cofactors are orderly recruited. To determine the best model, we proceeded with loss-of-function experiments for ERa and FOXA1 followed by ChIP of MED1 and SMC1A (Fig. 5). The same shRNA as previously described (Fig. 4 and Supplementary Fig. S3) were used. Molecularly, loss-of-function of the master transcription factor ERa and the pioneer transcription factor FOXA1 led to decreased recruitment of MED1 and SMC1A at enhancers and promoters of active genes *GREB1* (Fig. 5a and Supplementary Fig. S4) and *TTF1* (Fig. 5b and Supplementary Fig. S4). The total protein levels of the cofactors were not affected by the decreased expression of ERa and FOXA1 (Supplementary Fig. S4a). Therefore, we conclude that a combination of transcription factors is required to recruit and stabilize large protein complexes like Mediator and Cohesin to regulatory regions of actively transcribed regions.

## Discussion

While each cell presents unique biological characteristics, our results propose a shared common ground in transcriptional regulation. Indeed, Mediator, Cohesin and NIPBL occupy enhancer and promoter regions of actively transcribed genes in all primary^20^ and cancer cells (Fig. 1) tested so far. In addition, cancer cells are no exception to the simple rule stating that transcription factors provide cell specificity to the transcriptional program. Our results establish a model where a combination of FOXA and master transcription factors are part of the core transcriptional regulatory circuitry and essential to maintain the cancer cell properties (Figs 3 and 4). Regulatory regions are occupied by dozens and potentially more transcription factors in each cell^43^. Surprisingly, loss of a single transcription factor affected the recruitment of Mediator and Cohesin (Fig. 5) pointing toward a combinatorial effect in the stabilization and function of Mediator and Cohesin. This observation is corroborated by most reprogramming experiments where a combination of transcription factors is required to reach a new cell state^8^. However, we cannot exclude the possibility that the similar phenotypes observed in Mediator, Cohesin and transcription factor loss-of-function experiments (Figs 2 and 4) could be attributable to independent mechanisms including cell cycle defects. Therefore, our results suggest a non-redundant role of transcription factors at regulatory regions and postulate that FOXA and master transcription factors are required to stabilize large cofactor complexes like Mediator and Cohesin.

The identification of FOXA1 or FOXA2 as essential transcription factors in cancer cells (Figs 3 and 4) proposes the existence of a conserved role of the FOXA family in the core transcriptional regulatory circuitry. As pioneer transcription factors, FOXA1 and FOXA2 recognize their DNA motif even when the DNA is wrapped around nucleosomes^12^. This property could lead the way for master transcription factors to activate gene transcription. In hormone-dependent cancers, FOXA1 is essential to reprogram the genome-wide occupancy of the estrogen and androgen receptors and rewire the transcriptional program^44^,^45^. These observations suggest that FOXA transcription factors have the ability to direct binding of master transcription factors and to control the core transcriptional regulatory circuitry. Considering this central role of FOXA transcription factors, the successful development of specific inhibitors could lead to a new class of molecules to target cancer cells.

While key transcriptional regulators in breast adenocarcinoma (MCF7) and hepatocarcinoma (HEPG2) are known^14^,^36^,^45^, transcription factors maintaining lung adenocarcinoma (A549) are not well established. Integration of the genome-wide positioning of Mediator and Cohesin provide a valuable tool to predict key transcription factors in cell state maintenance. Indeed, our results suggest that the core transcriptional regulatory circuitry of A549 lung cancer cells is centered on FOSL2 and FOXA2 (Figs 3 and 4). Interestingly, both FOXA2 and FOSL2 are suspected to play major role in lung cancer progression. Indeed, FOSL2 is important to integrate the signaling through the TGF-beta/SMAD pathway and promote cellular migration of non-small cell lung adenocarcinomas^46^. Furthermore, decreased expression of FOXA2 following TGF-beta treatment leads to epithelial-to-mesenchymal transition^47^. Master transcription factors have been shown to determine the response to TGF-beta in various tissues through SMAD recruitment at regulatory regions^48^. One may speculate that decreased levels of FOXA2 allows the rewiring of the core transcriptional regulatory circuitry and free up FOSL2 for a role during the epithelial-to-mesenchymal transition. Therefore, the core transcriptional regulatory circuitry containing FOSL2 and FOXA2 could control the balance between maintenance of a lung epithelial *versus* mesenchymal phenotype.

The possibility of a general transcriptional mechanism centered on Mediator and Cohesin controlling the transcriptional program of normal and cancer cells raises questions regarding many human diseases. In addition to various types of cancers, genetic alterations in Mediator and Cohesin have been observed in developmental syndromes, neurological disorders and metabolic diseases^29^,^49^. For example, mutations of the cohesin subunit *STAG2* are frequent in various cancers^50^. Interestingly, evidence of aneuploidy are questioned and a transcription-dependent function is favored to explain the contribution of the Cohesin mutations toward cancer^28^,^51^. Similarly, MED12 is mutated at high frequencies in uterine leiomyomas and prostate cancers^52^,^53^. However, in uterine leiomyomas, all *MED12* mutations are found in exon 2 emphasizing alteration of a specific function of the Mediator subunit^52^. Additionally, during embryonic development, complete loss of Mediator and Cohesin are severely detrimental while missense mutations are less deleterious, but with complex tissue-specific phenotypes^29^,^49^. Taken together, these observations suggest that Mediator and Cohesin are potentially involved in a tissue-specific rather than a general transcriptional mechanism. Furthermore, the absence of genomic abnormalities in patients with Mediator and Cohesin mutations minimizes the possibility of cell cycle defects as the primary cause of these diseases. Therefore, we propose that modulation of the activity of the core transcriptional program is the central theme of many human diseases.

If transcriptional regulation is the primary cellular function of Mediator and Cohesin, their importance for other diseases is likely underestimated. Indeed, modulating the activity of regulatory regions is suspected to be an important mechanism leading to human diseases as SNPs are common in enhancer regions^24^,^25^. Variations in the sequence of an enhancer or promoter regions affect the binding affinity of the residing transcription factors^54^ which will result in differential recruitment of their associated cofactors including Mediator and Cohesin. Further experiments will demonstrate the importance of Mediator and Cohesin recruitment through FOXA and master transcription factors for many other human diseases.

## Methods

Full materials and methods are available in Supplementary Information.

### Cell Culture

MCF7 (ATCC, HTB-22) and HEPG2 (ATCC, HB-8065) were grown in DMEM (Gibco, 11965–092). A549 (ATCC, CCL-185) were grown in F12K medium (Gibco, 21127022). Culture medium were supplemented with 10% fetal bovine serum (Invitrogen, qualified 12483020), 100 μM MEM nonessential amino acids (Cellgro, 25–0250), 2 mM L-glutamine (Gibco, 25030–081), 100 U/ml penicillin, 100 μg/ml streptomycin (Gibco, 15170–063).

For ChIP experiments, MCF7 were kept the last 3 days into DMEM w/o phenol-red (Gibco, 31053–028) supplemented with 5% of Charcoal/Dextran treated FBS (Hyclone, AVH78911), 100 μM MEM nonessential amino acids (Cellgro, 25–0250), 2 mM L-glutamine (Gibco, 25030–081) and treated with 100 nM of Beta-Estradiol (SIGMA, E8875) for two hours.

### Chromatin Immunoprecipitation

ChIP-Seq were performed in biological duplicates as previously described^20^,^55^. Briefly, cells were crosslinked with 1% formaldehyde. Sonicated DNA fragments were immunoprecipitated with antibodies directed against MED1 (Bethyl Laboratories, A300-793A), SMC1A (Bethyl Laboratories, A300-055A), NIPBL, (Bethyl Laboratories, A301-779A) and ERa (Santa Cruz, sc-543x). ChIP-seq libraries and DNA sequencing were performed by the McGill University and Génome Québec Innovation Centre.

For single gene analysis, primers were designed in the predicted enhancers and promoters regions of *TFF1* and *GREB1* loci. For the *TFF1* locus, primers GAGATGATGCCACCGTACAC & CCCTCACTCACTTTGAAGCA were used for enhancer 1 (E1), ATCCAGTCCAGGAAGGAGGT & GTCAAGAGAGGAGGCTGTGG for enhancer 2 (E2) and CCGAGTCAGGGATGAGAGG & GGCCTCCTTAGGCAAATGTT for the TSS. For the *GREB1* locus, primers GAGCTGACCTTGTGGTAGGC & CAGGGGCTGACAACTGAAAT were used for enhancer 1 (E1), GGGATATGGCTTGTCCATTGT & CATGACACCAGGACCGTAAAG for enhancer 2 (E2) and ACCCAGCAAAACACTTCAGG & ATTCAGCAGTAGCCCTTCCA for the TSS. Two negative control regions (ATGTCAGGCCCATGAACGAT & GCATTCATGGAGTCCAGGCTTT and AGGACCTGCAGCAAACAGAA & TGTCTACATGGGCTAGTGTGCT) were used to calculate fold enrichments.

### Bioinformatic analyses

All ChIP-Seq reads (Supplementary Table S2) were aligned to the hg19 build of the human genome using the ChIP-Seq pipeline developed at McGill University and Génome Québec Innovation Centre (https://bitbucket.org/mugqic/mugqic_pipelinest). BigWig files were generated using Samtools 1.2^56^, bedtools 2.17.0^57^ and wigToBigWig (developed by the ENCODE team) with normalized count in millions of reads and a smoothing parameter of 225bp. Enriched regions were annotated using the UCSC database R package TxDb.Hsapiens.UCSC.hg19. KEGG pathways enrichments were assessed by hypergeometric tests. *In silico* motif searches were performed with the HOCOMOCO database using the PWMEnrich package. For overlaps with chromatin states and between ChIP-Seq datasets, the GenomicRanges R package was used.

### Lentiviral shRNAs

Plasmid catalog numbers and shRNA sequences are provided in Supplementary Table S3. Lentiviruses were produced as previously described^58^. Cells were transduced with the lentiviruses for 24 h before puromycin selection for the indicated time. Validation of efficiency were performed by Western blot (Supplementary Figs 1 and 3) using MED1 (Bethyl A300-793A, 1:5000), SMC1A (Bethyl, A300-055A, 1:5000), NIPBL, (Bethyl, A301-779A, 1:1000), ERa (Santa Cruz, sc-543x, 1:5000), FOXA1 (Santa Cruz, sc-101058 1:1000), FOXA2 (Abnova, 89-019-034, 1:1000), HNF4a (Santa Cruz, sc-8987x, 1:1000), FOSL2 (Santa Cruz, sc-604x, 1:1000), GAPDH (Pierce, PIMA515738, 1:25000) and Vinculin (SIGMA, V9131, 1:50000).

### Proliferation and colony formation assays

For proliferation and soft agar colony formation assays, cells were transduced with two shRNA per target with the exception of FOSL2 for which only one shRNA was efficiently knocking down all isoforms (Supplementary Fig. S3). Briefly, cells infected with lentiviral particles were selected for 24 hours prior to seeding. For proliferation assays, cell counts were measured using the Cytation 5 cell Imaging Multi-Mode Reader (BioTek Instruments) or the xCELLigence Real-Time Cell Analyzer (RTCA) instrument (ESBE Scientific and ACEA Biosciences) up to 8 days following seeding. Soft agar colony formation assays were performed as previously described^59^. Cells were maintained for 13 days (A549 and HEPG2) or 28 days (MCF7). Colonies were quantified using the ImageJ 1.46r software (NIH).

## Additional Information

**Accession codes**: The data sets supporting the results of this article are available in the NCBI’s Gene Expression Omnibus repository and are accessible through GEO Series accession number GSE76893 (http://www.ncbi.nlm.nih.gov/geo/query/acc.cgi?token=qrspsmoohdytnct&acc=GSE76893.

**How to cite this article**: Fournier, M. *et al*. FOXA and master transcription factors recruit Mediator and Cohesin to the core transcriptional regulatory circuitry of cancer cells. *Sci. Rep.* **6**, 34962; doi: 10.1038/srep34962 (2016).

## Supplementary Material

Supplementary Information
