# Supplementary material for: FOXA and master transcription factors recruit Mediator and Cohesin to the core transcriptional regulatory circuitry of cancer cells
Source: Sci Rep. 2016 Oct 14;6:34962. doi: 10.1038/srep34962 (PMC5064413; doi:10.1038/srep34962)
Supplement: Supplementary Information [file srep34962-s1.pdf]

Supplementary Information

FOXA and master transcription factors recruit Mediator and Cohesin to the core transcriptional regulatory circuitry of cancer cells

Michèle Fournier, Gaëlle Bourriquen, Fabien C. Lamaze, Maxime C. Côté, Éric Fournier, Charles Joly-Beauparlant, Vicky Caron, Stéphane Gobeil, Arnaud Droit and Steve Bilodeau

CONTENTS

Supplementary Tables

Supplementary Figures

Supplementary Data Files

Supplementary Experimental Procedures

- **Cell Culture Conditions**
- **Chromatin Immunoprecipitation**
  - *Antibodies*
  - *Immunoprecipitation*
  - *ChIP-qPCR (**Fig. 5 and Supplementary Figs S2 and S4**)*
  - *Library Preparation and Sequencing*
- **Bioinformatics and Analytical Methods**
  - *Short-Read pipeline processes*
  - *Data mining and parsing of the metadata*
  - *Gene annotation*
  - *Merging of ChIP-seq biological replicates*
  - *PCA analyses (**Fig. 1a**)*
  - *Browser tracks (**Figs 1b and 3c**)*
  - *Overlaps with chromatin states (**Fig. 1c,d**)*
  - Clustering of MED1, SMC1A (no CTCF), SMC1A (with CTCF) and NIPBL data ***(Fig. 1e)***
  - KEGG pathways ***(Fig. 2a)***
  - Overlaps with transcription factors ***(Fig. 3a)***
  - *DNA motif searches* ***(Fig. 3a)***
- **Lentiviral shRNAs**
- **Cell proliferation assays (Figs 2b and 4a)**
- **Soft-agar colony formation assays (Figs 2c and 4b)**

Supplementary References

Supplementary Tables

Supplementary Table S1. KEGG pathways enriched with genes occupied by MED1, SMC1A and NIPBL in MCF7, HEPG2 and A549 cells.

Supplementary Table S2. List of ChIP-Seq datasets used in the study.

Supplementary Table S3. List of shRNA plasmids and sequences used in the study

Supplementary Figures

Supplementary Fig. S1: shRNA targeting of MED1, SMC1A and NIPBL leads to decreased protein levels. Western blot validations of MED1, SMC1A and NIPBL depletions in MCF7, HEPG2 and A549 cells for the proliferation and colony formation assays used in **Fig. 2**. Vinculin was used as loading control to quantify the relative protein levels indicated below each panel. Two shRNAs were used per target and both were efficient at reducing protein levels compared to control shRNAs.

Supplementary Fig. S2: Validation of the core transcriptional regulatory circuitry in cancer cells. (a) Core transcriptional regulatory circuitry of MCF7, HEPG2 and A549 cells. The box represents the transcription factor gene loci while the oval represents the protein. Each line represents the interaction of a transcription factor with the indicated gene loci (-10kb to the end of the gene). **(b)** ChIP-qPCR validating the transcription factors occupancy at the core transcriptional regulatory circuitry genes. Regions with the most ChIP-Seq signal for each transcription factor were used for quantification. The bar graphs display enrichment fold calculated relative to control regions without ChIP-Seq signal and the error bars show the standard error of the mean for technical qPCR triplicates. **(c)** Gene expression changes following estrogen (E2, 100 nM) stimulation in MCF7. Fold changes were calculated relative to GAPDH at different time points. Error bars show the standard error of the mean for technical qPCR triplicates.

Supplementary Fig. S3: shRNA targeting of the identified transcription factors leads to decreased protein levels. (a) Western blot validations of the depletions of ERa and FOXA1 in MCF7 cells, HNF4A and FOXA2 in HEPG2 cells and FOSL2 and FOXA2 in A549 cells for the proliferation and colony formation assays used in **Fig. 4**. GAPDH or vinculin were used as loading control to quantify the relative protein levels indicated below each panel. Two shRNAs were used per target and both were efficient at reducing protein

levels compared to control shRNAs. It is to be noted that the shFOSL2-2 did not reduce the level of the all FOSL2 isoforms and was not used in **Fig. 4**.

Supplementary Fig. S4: Validation of the recruitment of Cohesin and Mediator by FOXA and master transcription factors. (a) Levels of MED1 and SMC1A are not affected by decreased expression of ERa and FOXA1 in MCF7 cells. Protein levels evaluated by Western blot for MED1 and SMC1A following shRNA targeting ERa and FOXA1 in MCF7 cells. Vinculin was used as loading control to quantify the relative protein levels indicated below each panel. Loss of ERa and FOXA1 do not significantly affect expression of MED1 and SMC1A. For **Fig. 5**, shESR1-1 and shFOXA1-2 were used since they were the most efficient. **(b)** Depletion of either the master transcription factor ERa or the pioneer transcription factor FOXA1 in MCF7 cells decreases MED1 and SMC1 recruitment at regions with low levels of ERa and FOXA1. Enrichment fold were calculated relative to control regions without MED1 or SMC1 signal. The bar graphs display the enrichment fold and error bars show the standard error of the mean for technical qPCR triplicates. **(c)** Cohesin subunits SMC3 and SA2 are recruited to the TSS of *GREB1* and *TFF1*. Enrichment fold measured by ChIP-qPCR were calculated relative to control regions without ChIP-Seq signal for Cohesin subunits. **(d)** Depletion of FOXA1 in MCF7 cells decreases SMC1A and SMC3 recruitment at the TSS of *GREB1* and *TFF1* suggesting the presence of the Cohesin complex.

Supplementary Data Files

Supplementary Data File 1 is a file containing all the ChIP-Seq enriched regions used in the study. The ZIP archive is accessible using the following URL: https://datahub-kazb7g4u.udes.genap.ca/Scientific_Reports/Supplementary_Data_Files/. (ZIP archive containing BED files; Size: 25.8 MB)

Supplementary Data File 2 is a file containing all the browser tracks used in the study**.** The ZIP file is accessible using the following URL: https://datahub-kazb7g4u.udes.genap.ca/Scientific_Reports/Supplementary_Data_Files/. (ZIP archive containing bigWig files; Size: 7.74 GB)

Supplementary Experimental Procedures

Cell Culture Conditions

MCF7 (ATCC, HTB-22), as well as HEPG2 (ATCC, HB-8065) were grown in DMEM (Gibco, 11965-092), while A549 (ATCC, CCL-185) were grown in F12K medium (Gibco, 21127022) as recommended by the supplier. All cell mediums were supplemented with 10% fetal bovine serum (Invitrogen, qualified 12483020), 100 μM MEM nonessential amino acids (Cellgro, 25-0250), 2 mM L-glutamine (Gibco, 25030-081), 100 U/ml penicillin, 100 μg/ml streptomycin (Gibco, 15170-063). For ChIP experiments, MCF7 were kept in DMEM w/o phenol-red (Gibco, 31053-028) supplemented with 5% of Charcoal/Dextran treated FBS (Hyclone, AVH78911), 100 μM MEM nonessential amino acids (Cellgro, 25-0250) and 2 mM L-glutamine (Gibco, 25030-081) for 3 days prior to 100nM of Beta-Estradiol (SIGMA, E8875) for two hours.

Chromatin Immunoprecipitation

Antibodies

For MED1 (CRSP1/TRAP220) occupied genomic regions, we performed the ChIP-Seq experiments using the Bethyl Laboratories (A300-793A) antibody. The affinity purified antibody was raised in rabbit against an epitope corresponding to amino acids 1523-1281 mapping at the C-terminus of human MED1. Specificity of the antibody for ChIP-Seq experiments was validated previously [1].

For SMC1A occupied genomic regions, we performed the ChIP-Seq experiments using the Bethyl Laboratories (A300-055A) affinity purified rabbit polyclonal antibody. The epitope recognized by A300-055A maps to a region between residue 1175 and the C-terminus of human SMC1A. Specificity of the antibody for ChIP-Seq experiments was validated previously [1].

For NIPBL occupied genomic regions, we performed the ChIP-Seq experiments using the Bethyl Laboratories (A301-779A) affinity purified rabbit polyclonal antibody. The affinity purified antibody was raised in rabbit to a region between amino acid residues 1025 and 1075 of human NIPBL. Specificity of the antibody for ChIP-Seq experiments was validated previously [1].

For ERa occupied genomic regions, we performed the ChIP-Seq experiments using the Santa Cruz (sc-543x) affinity purified rabbit polyclonal antibody. The affinity purified antibody was raised in rabbit to a region between amino acid residues 1025 and 1075 of human ERa. Specificity of the antibody for ChIP-Seq experiments was validated previously by ENCODE project consortium (https://genome.ucsc.edu/ENCODE/validation/antibodies/human_ERalpha_a_validation_Myers.pdf).

Immunoprecipitation

ChIP (ChIP-qPCR) and ChIP coupled with massively parallel sequencing (ChIP-seq) were performed as previously described [1,2]. Briefly, cells were crosslinked with 1% formaldehyde in culture medium for 10 minutes at room temperature. Cells were then lysed in 10 ml lysis buffer (0.1%SDS, 1% Triton X-100, 2mM EDTA, 20mM Tris-HCl ph 8.0 and 150 mM NaCl) and chromatin was sheared to 200-600 bp fragments using a Bioruptor Sonicator (Diagenode). Chromatin extract from $5x10^7$ cells was incubated overnight at 4^oC with 100 ul of Dynabeads Protein G magnetic beads (Life Technologies, 10004D) previously incubated with 10 ug of selected antibodies (NIPBL (Bethyl, A301-779A), MED1 (Bethyl, A300-793A), SMC1A (Bethyl, A300-055A), ERa (Santa Cruz, sc543X), SMC3 (Bethyl, A300-158A) and SA2 (Bethyl, A300-060A)).

*ChIP-qPCR (**Fig. 5 and Supplementary Figs S2 and S4**)*

For single gene analysis, primers were designed in the predicted enhancers and promoters regions of *TFF1* and *GREB1* genes. For the *TFF1* locus, primers GAGATGATGCCACCGTACAC and CCCTCACTCACTTTGAAGCA were used for E1, ATCCAGTCCAGGAAGGAGGT and GTCAAGAGAGGAGGCTGTGG for E2 and CCGAGTCAGGGATGAGAGG and GGCCTCCTTAGGCAAATGTT for the TSS. For the *GREB1* locus, primers GAGCTGACCTTGTGGTAGGC and CAGGGGCTGACAACTGAAAT were used for E1, GGGATATGGCTTGTCCATTGT and CATGACACCAGGACCGTAAAG for E2 and ACCCAGCAAAACACTTCAGG and ATTCAGCAGTAGCCCTTCCA for the TSS. Two negative control regions (ATGTCAGGCCCATGAACGAT & GCATTCATGGAGTCCAGGCTTT and AGGACCTGCAGCAAACAGAA & TGTCTACATGGGCTAGTGTGCT) were used to calculate fold enrichments.

For supplementary Figs S2 and S4, the following primers were used:

ChIP-qPCR		
**Target**	**Forward**	**Reverse**
**MCF7**		
ESR1	CTGCAGTAGGCACTCAGTAAAT	TCAAACTAACCTGAAACTCGGT
FOSL2	GCAGGCACTGGGAACAG	CACAGAGGGTCTCCCTATCTC
FOXA1	CACAGCACCGGTTTATATCTTTATG	CTGCTCTCAGTTTCTTCCTCTT
JUND	CGGTAAACGCCACAACAG	ATGACGTCAACCCACAAGG
Low ERa	GCAACACCAAACACTGCTATC	TGTTTCTCGAGGAGTATATCTGAAC
Low FOXA1	GGTAGGATACCACAGGCTGATA	AATGCTGCGGAACGAGTC
**HEPG2**		
CEBPB (ChIP FOXA)	CTCTCTGCTTCTCCCTCTGC	TAAGCGATTACTCAGGGCCC
CEBPB (ChIP HNF4A)	CTGGGCCAGTGTTTTATCCC	GTTATCTGCTGAGCTGGGGT
FOXA1 (ChIP FOXA)	GGTTTCCGAGGAAGGGATTA	CCGGGACCTAAAAGTCAACA
FOXA2 (ChIP FOXA)	CCCCTCCCTGTTACAGTTCA	GGTGTCTGAGGAGTCGGAGA
FOXA2 (ChIP HNF4A)	CTATTTTGCTCCTGGGTGGA	AAAGTCCGCTCCTTGGAAGT
HNF4A	TTCTCCTGGCTCTGACACTG	CAACAGTCTGCTTGTTCCCC
**A549**		
ATF3	TAGCATTACGTCAGCCTGGG	GCGTTGCATCACCCCTTTTA
FOSL2 (ChIP FOSL2)	CAAGGTGGAGTCTGGGGTTG	TCTGCCCGAGATGAGTCACT
FOSL2 (ChIP FOXA)	AGTTTTCTGAGTTTTGCTGCTG	TTTACGCGCCTCTCCCAG
FOXA1 (ChIP FOSL2)	AGAGCTTCAGACTAGAAAAGGGA	GCCTAACGTCACTTGGCTC
FOXA1 (ChIP FOXA)	CAAGCCCACGCCTTTATATT	GACCTCGGAAGGACATCAAA
FOXA2 (ChIP FOSL2)	ACACCACCTTTTCCTTGCAA	TTGGGGATAGGGAAGCAGAG
FOXA2 (ChIP FOXA)	CTTTTCAGTCACAACCGAGGT	TCTTCTCTCCTCACCCCTCC
JUND	AGTTGTTGGGGAGAGAGGAC	TTTACTGCGACTTCCCTCCC
**RTqPCR**		
ESR1	AGCTCCTCCTCATCCTCC	ATAGAGGGGCACCACGTTCT
FOXA1	GTGAAGATGGAAGGGCATGA	CCAGGCCTGAGTTCATGTT
JUND	ACGAGCTCACAGTTCCTCTA	GCTGGTTCTGCTTGTGTAAATC
FOSL2	CCTGGCGTGATCAAGACCAT	CGACGCTTCTCCTCCTCTTC

Library Preparation and Sequencing

ChIP-seq libraries and DNA sequencing were performed by the McGill University and Génome Québec Innovation Centre (MUGQIC). Briefly, ChIP libraries were prepared with the TruSeq DNA sample prep kit using indexing adapters according to the manufacturer's recommendations (Illumina). The starting amount of ChIP DNA used for library preparation ranged from 2.5 ng to 10.5 ng per sample. Library quality and average fragment size was confirmed with Bioanalyzer DNA analysis chips (25-1000 bp, Agilent). TruSeq libraries were multiplexed on the Illumina HiSeq2000. We used single-end reads of 50 bp. Two biological replicates were performed for each target.

Bioinformatics and Analytical Methods

All the ChIP-Seq datasets used in the study (**Supplementary Table S2**) were analyzed with ChIP-Seq pipeline developed at the MUGQIC (version 2.1.0) which is briefly summarized below:

Short-Read pipeline processes

All samples were processed using Trimmomatic 0.33 [3] with the following parameters: LEADING=30, TRAILING=30, SLIDINGWINDOW=4:30, MINLEN=30. ChIP-seq reads were mapped against the hg19 build of the human reference genome using BWA 0.7.12 [4] with default parameters. MACS2 2.1.0 [5] with default parameters was used for peak calling. All replicates (two replicates for our ChIP-Seq experiments and up to eight for ENCODE datasets) were processed separately until this step.

Data mining and parsing of the metadata

ENCODExplorer

(https://www.bioconductor.org/packages/release/bioc/html/ENCODExplorer.html) was used to parse the metadata and retrieve the Fastq files from the ENCODE consortium. ChIPseeker [6] was used for data mining of data deposited in the GEO database.

Gene annotation

All peak regions defined by MACS2 were annotated using the UCSC database R package TxDb.Hsapiens.UCSC.hg19.knownGene

(http://bioconductor.org/packages/release/data/annotation/html/TxDb.Hsapiens.UCSC.hg19.knownGene.html) and ChIPseeker.

Merging of ChIP-seq biological replicates ConsensusSeekeR (https://www.bioconductor.org/packages/devel/bioc/html/consensusSeekeR.html) was used to merge the narrowPeak files generated by MACS2 for all replicates to find the best consensus at a given genomic regions. The following parameters were used: extendingSize = 200, expandToFitPeakRegion = TRUE, shrinkToFitPeakRegion = TRUE. The extendingSize parameter allows to determine the size of padding on both sides of the position of the peaks median to create the consensus region. The expandToFitPeakRegion parameter allows to extend the consensus regions to include the entire narrow peak regions of all peaks included in the unextended consensus region. shrinkToFitPeakRegion allow to shrink the consensus regions size to fit the narrow peak regions of the peaks when all those regions are smaller than the consensus region. The minimum number of replicates with a significant enriched region (minNbrExp) was set to 2 out 3 for targets with 3 and more datasets. minNbrExp was set to 2 for targets with 2 biological replicates.

*PCA analysis (**Fig. 1a**)*

A binary matrix encoding the presence or absence of NIPBL, MED1, SMC1A or CTCF at all genomic loci where at least one factor was present was constructed (Rows: loci; Columns: ChIP-Seq targets). Principal component analysis (PCA) was performed using the R package FactoMineR (http://CRAN.R-project.org/package=FactoMineR) considering the presence or absence of NIPBL, MED1, SMC1A or CTCF as the variables. Visualization of the variables on the first two components was performed using a "correlation circle plot" with the factoextra R package (https://github.com/kassambara/factoextra). For the representation, the correlation/loading values are on the 0 to 1 scale, with a value of 1 meaning 100% correlation/loading with the dimension. The density of loci was calculated as the projection of the loci coordinates onto the first dimension (which discriminated the most the loci with CTCF and without CTCF). The y-axis is scaled to one unit and the x-axis is an arbitrary unit representing the coordinates of the loci on the first dimension. Each group (No signal, SMC1A (no CTCF), and SMC1A (with CTCF)) was assigned a color.

*Browser tracks (**Figs 1b and 3c**)*

BigWig files were generated by using Samtools 1.2 [4], bedtools 2.17.0 [7] and wigToBigWig (developed by the ENCODE team) with normalized count in millions of reads and a smoothing parameter of 225 bp.

*Overlaps with chromatin states (**Fig. 1c,d**)*

The 18 chromatin states for HEPG2 and A549 were retrieved from the consortium Epigenomic Roadmap. The chromatin states were generated with ChromHMM [8,9] with a core set of 6 chromatin marks (H3K4me3, H3K4me1, H3K36me3, H3K27me3, H3K27ac, H3K9me3). Abbreviations: 1_TssA (Active TSS), 2_TssFlnk (Flanking TSS), 3_TssFlnkU (Flanking TSS Upstream), 4_TssFlnkD (Flanking TSS Downstream), 5_Tx (Strong transcription), 6_TxWk (Weak transcription), 7_EnhG1 (Genic enhancer 1), 8_EnhG2 (Genic enhancer 2), 9_EnhA1 (Active Enhancer 1), 10_EnhA2 (Active Enhancer 2), 11_EnhWk (Weak Enhancer), 12_ZNF/Rpts (ZNF genes & repeats), 13_Het (Heterochromatin), 14_TssBiv (Bivalent/Poised TSS), 15_EnhBiv (Bivalent Enhancer), 16_ReprPC (Repressed PolyComb), 17_ReprPC (Weak Repressed PolyComb), 18_Quies (Quiescent/Low).

For MCF7, the same 6 chromatin marks were used (H3K4me3, H3K4me1, H3K36me3, H3K27me3, H3K27ac, H3K9me3) as follow. Regions enriched for H3K27ac and H3K4me3 were considered Active TSS (TssA). Regions enriched with H3K36me3 were considered transcriptionally active regions (Tx). Regions enriched with both H3K27ac and H3K4me1 were considered active enhancers (EnhA). Regions enriched with H3K9me3 were considered as heterochromatin (Het). Regions enriched for H3K4me3 and H3K27me3, but not H3K27ac were considered Bivalent/Poised TSS (TssBiv). Regions enriched for H3K4me1 and H3K27me3, but not H3K27ac were considered Bivalent Enhancers (EnhBiv). Regions enriched for H3K27me3, but not H3K27ac, TssBiv or EnhBiv were considered Repressed PolyComb (ReprPC). Abbreviations: 1_TssA (Active TSS), 2_Tx (Strong transcription), 3_EnhA (Active Enhancer), 4_Het (Heterochromatin), 5_TssBiv (Bivalent/Poised TSS), 6_EnhBiv (Bivalent Enhancer), 7_ReprPC (Repressed PolyComb).

Once the chromatin states were defined, the ratio of nucleotides from each chromatin state overlapping the consensus peak regions of NIPBL, MED1, SMC1A (with and without

CTCF) was calculated. Additionally, that same ratio was calculated for regions where the consensus peaks of NIPBL, MED1 and SMC1A intersected.

Clustering of MED1, SMC1A (no CTCF), SMC1A (with CTCF) and NIPBL data ***(Fig. 1e)***

All MED1, NIPBL, SMC1A (with CTCF) and SMC1A (no CTCF) regions from all cell lines were converted into a binary presence matrix, as in **Fig. 1a**. The overlap in-between all region categories was calculated using the category with the lowest number of regions as the denominator. The resulting matrix was subjected to hierarchical clustering using euclidian distance and Unweighted Pair Group Method with Arithmetic Mean (UPGMA).

KEGG Pathways ***(Fig. 2a)***

For each cell line, the intersections of the consensus regions for MED1, NIPBL and SMC1A were computed. The selected regions were then annotated using the TxDb.Hsapiens.UCSC.hg19.knownGene package (http://bioconductor.org/packages/release/data/annotation/html/TxDb.Hsapiens.UCSC.hg19.knownGene.html). Comparison to the hypergeometric distribution was used to evaluate enrichment of all KEGG pathways within the set of identified genes. All generated p-values were then subjected to a unified FDR correction.

Overlaps with transcription factors ***(Fig. 3a)***

The overlap between the intersection of the MED1, NIPBL, and SMC1A consensus regions and the consensus regions of all available ENCODE factors were computed. The reported pairwise overlap is:

$$\mathrm{Overlap_{TF}} = \frac{\mathrm{N_{NMS,TF}}}{min(\mathrm{N_{NMS}}, \mathrm{N_{TF}})}$$

Where $N_{NMS,TF}$ is the number of regions where NIPBL, MED1, SMC1A and the ENCODE TF are all present, N_{NMS} is the number of regions where NIPBL, MED1, SMC1A are present, and N_{TF} is the number of regions where the ENCODE TF is present.

DNA motif searches ***(Fig. 3a)***

The regions bearing NIPBL, MED1 and SMC1A were scanned for known human transcription factor binding site models from the HOCOMOCO v9 [10] database using the

Bioconductor package PWMEnrich (https://www.bioconductor.org/packages/release/bioc/html/PWMEnrich.html).

Lentiviral shRNAs

Lentiviruses were produced according to the TRC1.5 system from Sigma and plasmids and shRNA sequences used are shown in the **Supplementary Table S3**. The packaging cell line HEK293T was used to produce lentiviral particles containing the pLKO.1 vectors. Cells were transfected with 1:3:6 µg DNA respectively composed of packaging vectors pMD2.G (Addgene, #12259) and psPAX2 (Addgene, #12260), as well as the specific shRNA constructs. Transfections were performed with X-tremGENE 9 transfection reagent (Roche), according to manufacturer's instructions. Supernatants were harvested 48h following transfection. Supernatants were filtered and added to recipient cell lines with 8 µg/mL polybrene (SIGMA, H9268), as described on Addgene website. Cells infected with the lentivirus were incubated 24h before selection with puromycin for 48 h. To assess the efficiency of each shRNA, Western blot analyses were performed using antibodies against NIPBL, (Bethyl, A301-779A, 1:1000), MED1 (Bethyl A300-793A, 1:5000), SMC1A (Bethyl, A300-055A, 1:5000), ERa (Santa Cruz, sc-543x, 1:5000), FOXA1 (Santa Cruz, sc-101058, 1:1000), FOXA2 (Abnova, 89-019-034, 1:1000), HNF4A (Santa Cruz, sc-8987x, 1:1000), FOSL2 (Santa Cruz, sc-604x, 1:1000), GAPDH (Pierce, PIMA515738, 1:25000) and Vinculin (SIGMA, V9131, 1:50000)..

Cell proliferation assays (*Figs 2b and 4a*)

Cell proliferation assays were performed on A549, HEPG2 and MCF7 with cells infected with lentiviruses containing the validated shRNA constructs. For the Cytation 5 cell Imaging Multi-Mode Reader (BioTek Instruments), A549 ($5X10^3$ cells/well) were seeded into black-sided clear bottom 96-well plates (Corning, 3603), while HEPG2 ($1.2X10^5$ cells/well) and MCF7 ($1.5X10^4$ cells/well) were seeded into clear bottom 12-well plates (Corning, 3513), following a 24h selection. Cells were stained with Hoechst 33342 at 10µg/ml in media for 30 min, at 37°C, prior to imaging. Cell proliferation was measured up to 8 days after seeding. All cell nucleus counting experiments were performed in biological duplicates or triplicates (using 16 fields/well with a 4X microscope objective. Threshold was set to 8,000 with a minimum object size of 5 µm and a maximum size set to 100 µm.

For the xCELLigence Real-Time Cell Analyzer (RTCA) instrument (ESBE Scientific and ACEA Biosciences), MCF7 ($1.5X10^4$ cells/well) were seeded into E-Plate VIEW 16 PET, a specialized 16-well plate used with the RTCA instrument, into selection media. The cell proliferation index was measured using impedance up to 8 days after seeding.

Soft agar colony formation assays (Figs 2c and 4b)

Soft agar colony formation assays were performed as previously described [11]. MCF7, HEPG2 and A549 cells infected with lentiviruses containing the validated shRNA constructs and selected for 24 hours were used. Two days after their infection, cells were seeded in complete growth medium with 0.3% agar layered onto 0.5% agar beds in 6-well plates (A549: $2X10^4$ cells/well, HEPG2: $2X10^4$ cells/well and MCF7: $6X10^4$ cells/well). Complete medium was added on top twice a week for 13 days (A549 and HEPG2) or 28 days (MCF7). Colonies larger than $800um^2$ were counted as positive for growth with ImageJ 1.46r software (NIH).

Supplementary References

1. Kagey, M. H. *et al.* Mediator and cohesin connect gene expression and chromatin architecture. *Nature* **467,** 430–5 (2010).
2. Bilodeau, S., Kagey, M. H., Frampton, G. M., Rahl, P. B. & Young, R. A. SetDB1 contributes to repression of genes encoding developmental regulators and maintenance of ES cell state. *Genes Dev.* **23,** 2484–9 (2009).
3. Bolger, A. M., Lohse, M. & Usadel, B. Trimmomatic: a flexible trimmer for Illumina sequence data. *Bioinformatics* **30,** 2114–2120 (2014).
4. Li, H. *et al.* The Sequence Alignment/Map format and SAMtools. *Bioinformatics* **25,** 2078–2079 (2009).
5. Zhang, Y. *et al.* Model-based Analysis of ChIP-Seq (MACS). *Genome Biol.* **9,** R137 (2008).
6. Yu, G., Wang, L.-G. & He, Q.-Y. ChIPseeker: an R/Bioconductor package for ChIP peak annotation, comparison and visualization. *Bioinformatics* **31,** 2382–2383 (2015).
7. Quinlan, A. R. & Hall, I. M. BEDTools: a flexible suite of utilities for comparing genomic features. *Bioinformatics* **26,** 841–842 (2010).

8. Ernst, J. *et al.* Mapping and analysis of chromatin state dynamics in nine human cell types. *Nature* **473,** 43–9 (2011).
9. Kundaje, A. *et al.* Integrative analysis of 111 reference human epigenomes. *Nature* **518,** 317–330 (2015).
10. Kulakovskiy, I. V *et al.* HOCOMOCO: a comprehensive collection of human transcription factor binding sites models. *Nucleic Acids Res.* **41,** D195–202 (2013).
11. Oster, S. K., Mao, D. Y. L., Kennedy, J. & Penn, L. Z. Functional analysis of the N-terminal domain of the Myc oncoprotein. *Oncogene* **22,** 1998–2010 (2003).

Fournier_Supplementary Figure S1

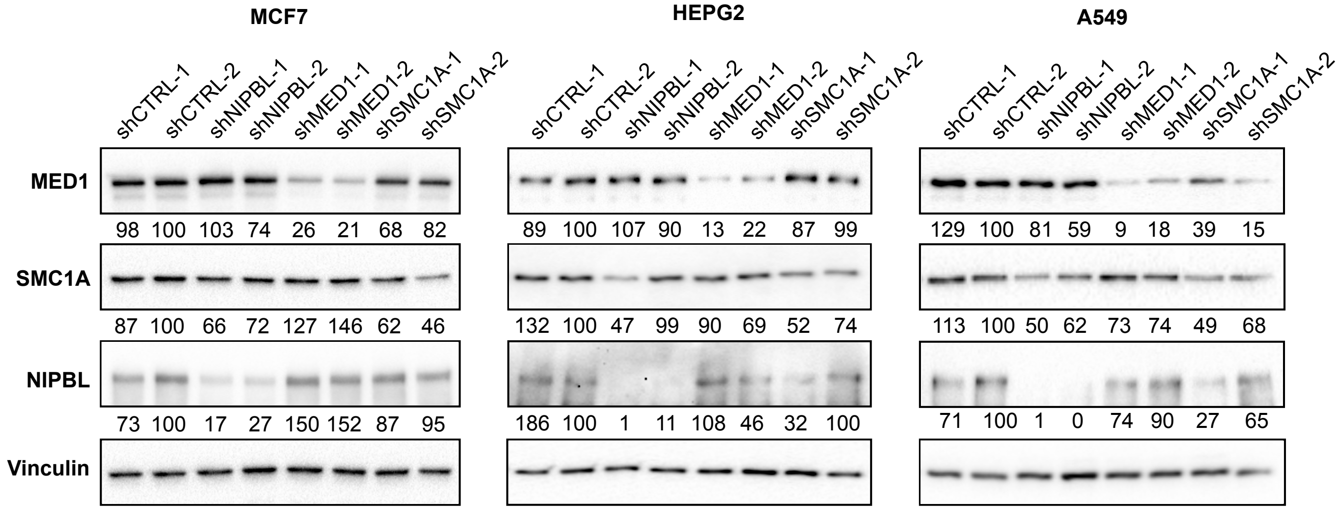

Fournier_Supplementary Figure S2

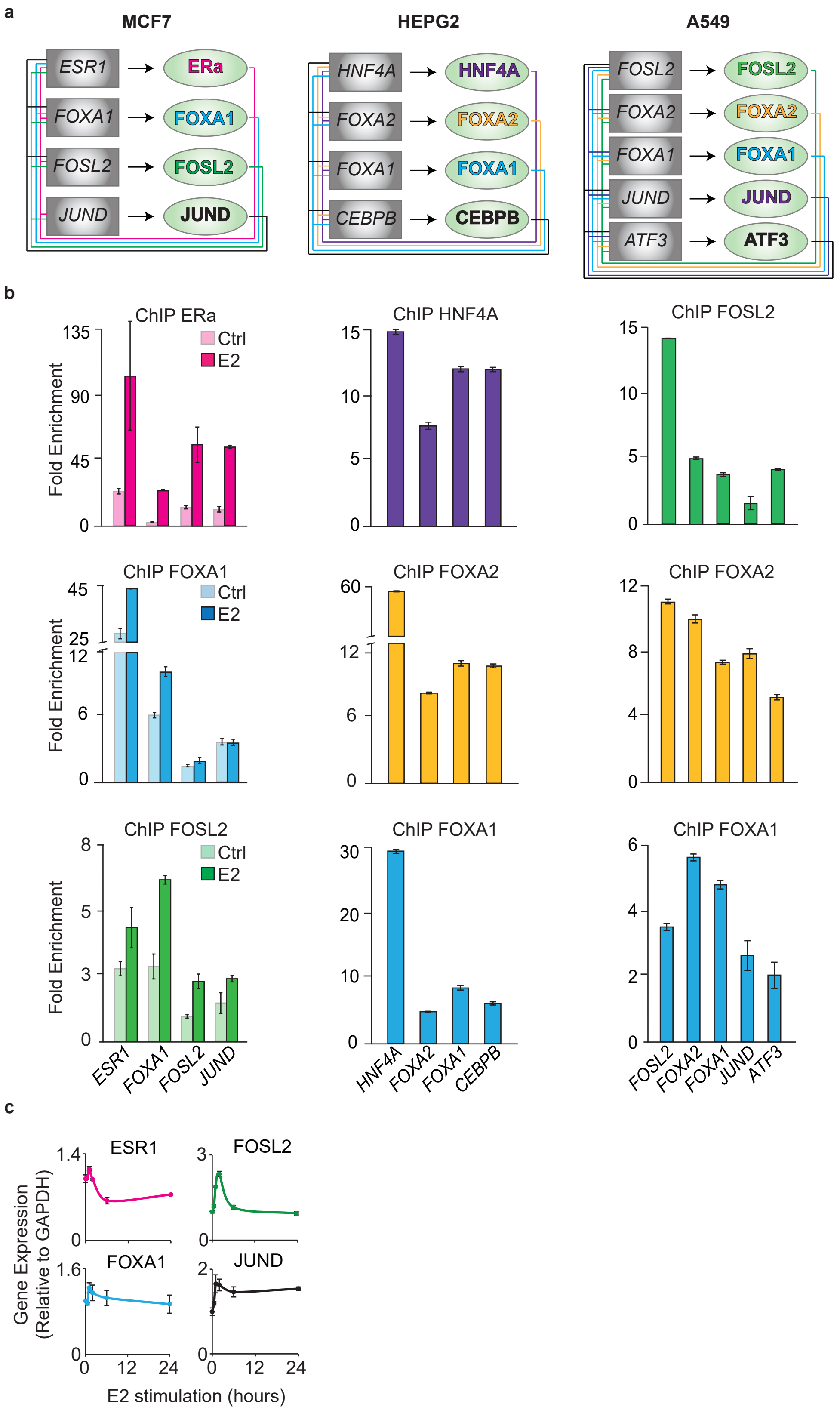

Fournier_Supplementary Figure S3

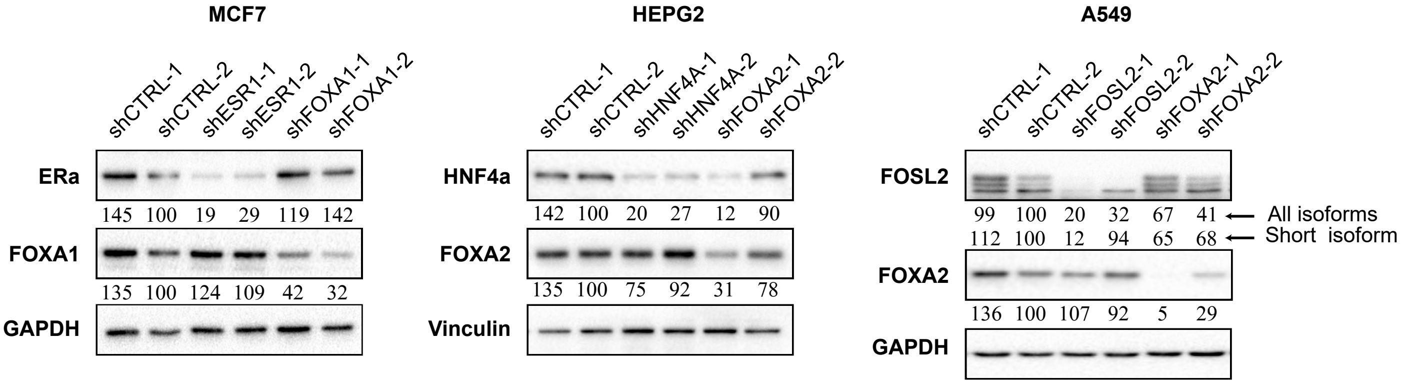

Fournier_Supplementary Figure S4

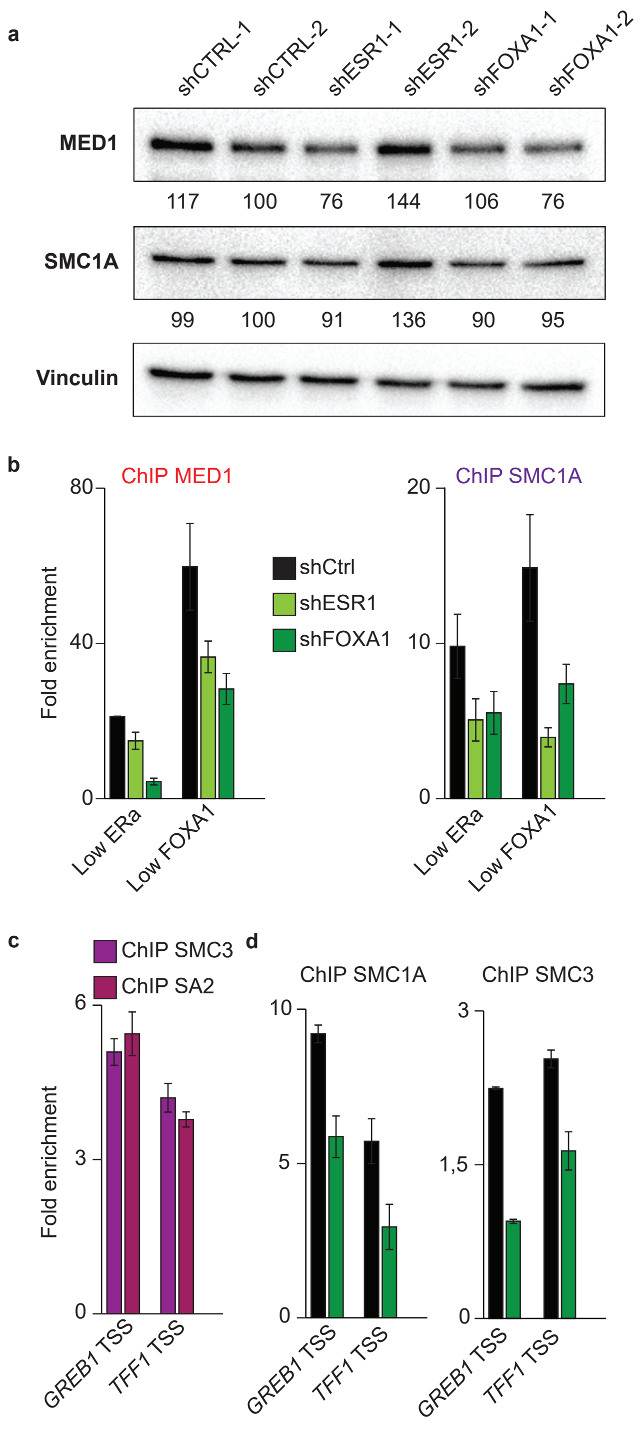

Supplementary Table S1 – KEGG pathways enriched with genes occupied by MED1, SMC1A and NIPBL in MCF7, HEPG2 and A549 cells.

	-log10 (FDR corrected p-value)		
	HEPG2	MCF7	A549
hsa00970 Aminoacyl-tRNA biosynthesis	0.06448989	0.1352676	0.0173519
hsa02010 ABC transporters	0.88476264	0.45408152	1.80907079
hsa03008 Ribosome biogenesis in eukaryotes	0.03666085	0.08763324	0.00549542
hsa03010 Ribosome	0.0081719	0.03666085	0.00526644
hsa03013 RNA transport	0.0037942	0.01820138	0.01809528
hsa03015 mRNA surveillance pathway	0.13116847	0.08333723	0.03204529
hsa03018 RNA degradation	0.04760122	0.29100893	0.36194062
hsa03020 RNA polymerase	0.18573026	0.26993475	0.0914896
hsa03022 Basal transcription factors	0.31232912	0.45408152	0.04760122
hsa03030 DNA replication	0.16345299	0.24263714	0.23081073
hsa03040 Spliceosome	0.04760122	0.03778088	0.00549542
hsa03050 Proteasome	0.31428034	0.20452893	0.04760122
hsa03060 Protein export	0.24263714	0.31428034	0.65354423
hsa03320 PPAR signaling pathway	3.37498227	0.31428034	0.294212
hsa03410 Base excision repair	0.18047004	0.26152946	0.08690141
hsa03420 Nucleotide excision repair	0.30530343	0.44019414	0.30530343
hsa03430 Mismatch repair	0.24263714	0.31428034	0.35364118
hsa03440 Homologous recombination	0.45408152	0.28263629	0.29100893
hsa03450 Non-homologous end-joining	0.33263641	0.42695714	0.24837149
hsa03460 Fanconi anemia pathway	0.27876652	0.72059919	0.27109174
hsa04010 MAPK signaling pathway	1.03566082	0.70771226	1.13728408
hsa04012 ErbB signaling pathway	0.43715382	0.45121207	1.05343614
hsa04014 Ras signaling pathway	0.84592124	0.31428034	1.08501418
hsa04015 Rap1 signaling pathway	0.44925419	1.34726651	2.23139097
hsa04020 Calcium signaling pathway	0.79585701	0.45121207	1.07781407
hsa04022 cGMP-PKG signaling pathway	1.08501418	1.20476898	0.43052127
hsa04024 cAMP signaling pathway	0.36194062	0.98704607	0.74617128
hsa04060 Cytokine-cytokine receptor interaction	0.79898036	0.24837149	1.80907079
hsa04062 Chemokine signaling pathway	1.3041869	0.8281362	0.32561133
hsa04064 NF-kappa B signaling pathway	0.85891916	0.08333723	0.78294821
hsa04066 HIF-1 signaling pathway	1.90543498	0.61199009	1.80907079
hsa04068 FoxO signaling pathway	1.383675	0.44053573	1.20699442
hsa04070 Phosphatidylinositol signaling system	0.80134283	0.9119735	0.51172707
hsa04071 Sphingolipid signaling pathway	0.42747802	0.5101745	0.16615426
hsa04080 Neuroactive ligand-receptor interaction	0.05611979	0.62106254	0.38551948
hsa04110 Cell cycle	0.40622927	0.30530343	0.32608645
hsa04114 Oocyte meiosis	0.19172148	0.55697608	0.29100893
hsa04115 p53 signaling pathway	0.36194062	1.20699442	0.87162763
hsa04120 Ubiquitin mediated proteolysis	0.23360708	0.27899562	0.28263629
hsa04122 Sulfur relay system	0.88476264	0.46800068	0.29100893
hsa04130 SNARE interactions in vesicular transport	0.17428611	0.25265786	0.70791776
hsa04140 Regulation of autophagy	0.14722851	0.22553797	0.0628508
hsa04141 Protein processing in endoplasmic reticulum	0.35364118	0.32091736	0.31428034
hsa04142 Lysosome	0.28263629	0.16615426	0.03301846
hsa04144 Endocytosis	0.53353389	0.36194062	0.83877231
hsa04145 Phagosome	0.42414457	0.23500199	0.21447197
hsa04146 Peroxisome	1.20699442	0.09783438	0.11910916
hsa04150 mTOR signaling pathway	0.67680873	0.35714788	0.54287035
hsa04151 PI3K-Akt signaling pathway	0.36194062	0.56704944	1.64368251
hsa04152 AMPK signaling pathway	0.59038668	0.30530343	0.96434529
hsa04210 Apoptosis	1.20537507	0.27109174	0.84629036
hsa04260 Cardiac muscle contraction	0.16779307	0.80197349	0.36194062
hsa04261 Adrenergic signaling in cardiomyocytes	0.30727917	0.82766809	0.31428034
hsa04270 Vascular smooth muscle contraction	0.83098243	1.30206728	0.82766809
hsa04310 Wnt signaling pathway	1.30206728	0.27109174	1.81815184
hsa04320 Dorso-ventral axis formation	1.80907079	0.65928509	1.20699442
hsa04330 Notch signaling pathway	0.2970733	0.43420076	0.72059919
hsa04340 Hedgehog signaling pathway	0.28428077	0.18261675	0.44019414
hsa04350 TGF-beta signaling pathway	0.69026265	1.05913914	2.93344883
hsa04360 Axon guidance	0.7819282	0.70924373	1.30206728
hsa04370 VEGF signaling pathway	0.4160026	0.35364118	0.75842459
hsa04380 Osteoclast differentiation	1.20476898	0.93889294	1.07781407
hsa04390 Hippo signaling pathway	1.20537507	1.62576397	3.25432318
hsa04510 Focal adhesion	0.8281362	0.75705689	3.06722551
hsa04512 ECM-receptor interaction	0.15462351	1.07781407	1.09981997
hsa04514 Cell adhesion molecules (CAMs)	0.32374736	0.4160026	0.45408152
hsa04520 Adherens junction	1.08501418	0.30530343	3.53315075

hsa04530 Tight junction	0.12782002	0.27876652	0.98704607
hsa04540 Gap junction	0.65354423	0.44376705	0.30530343
hsa04550 Signaling pathways regulating pluripotency c	2.40080884	0.85725599	3.25432318
hsa04610 Complement and coagulation cascades	2.42901562	0.13116847	0.64253303
hsa04611 Platelet activation	1.05343614	0.75193439	0.33844188
hsa04612 Antigen processing and presentation	0.04760122	0.11215822	0.00927162
hsa04614 Renin-angiotensin system	0.95348518	0.31428034	0.65354423
hsa04620 Toll-like receptor signaling pathway	0.20675133	0.36194062	0.21993112
hsa04621 NOD-like receptor signaling pathway	0.44053573	0.16118378	0.24009169
hsa04622 RIG-I-like receptor signaling pathway	0.35338791	0.31279641	0.06634004
hsa04623 Cytosolic DNA-sensing pathway	0.21993112	0.13839477	0.19890669
hsa04630 Jak-STAT signaling pathway	0.75193439	0.35516932	1.30206728
hsa04640 Hematopoietic cell lineage	0.43715382	0.26349006	0.20093494
hsa04650 Natural killer cell mediated cytotoxicity	0.35714788	0.28428077	0.02137295
hsa04660 T cell receptor signaling pathway	0.75193439	0.86653541	0.1352316
hsa04662 B cell receptor signaling pathway	0.82432591	0.84880426	0.28263629
hsa04664 Fc epsilon RI signaling pathway	0.60457038	0.59038668	0.66590546
hsa04666 Fc gamma R-mediated phagocytosis	1.09912109	0.98308861	0.97054854
hsa04668 TNF signaling pathway	0.9119735	0.56704944	2.38790137
hsa04670 Leukocyte transendothelial migration	0.43613898	0.31596355	1.20699442
hsa04672 Intestinal immune network for IgA productior	0.30530343	0.19464349	0.04385493
hsa04710 Circadian rhythm	0.78294821	0.27356757	0.46967574
hsa04713 Circadian entrainment	0.38551948	0.95348518	0.37807177
hsa04720 Long-term potentiation	0.61540791	0.32561133	0.45582565
hsa04721 Synaptic vesicle cycle	0.06959479	0.34168589	0.32608645
hsa04722 Neurotrophin signaling pathway	0.61894828	0.31428034	0.16615426
hsa04723 Retrograde endocannabinoid signaling	0.22541751	0.62681662	0.34331287
hsa04724 Glutamatergic synapse	0.87162763	1.33573973	0.53683756
hsa04725 Cholinergic synapse	0.46505437	0.82766809	0.11477478
hsa04726 Serotonergic synapse	0.19172148	0.19172148	0.40123762
hsa04727 GABAergic synapse	0.43420076	1.02696491	0.43676602
hsa04728 Dopaminergic synapse	0.3741105	0.29235464	0.13534378
hsa04730 Long-term depression	0.42414457	0.35714788	0.77539653
hsa04740 Olfactory transduction	0.00064792	0.00927162	0.00193814
hsa04742 Taste transduction	0.10117202	0.40667886	0.27876652
hsa04744 Phototransduction	0.20452893	0.28489626	0.29841977
hsa04750 Inflammatory mediator regulation of TRP cha	1.05343614	1.20699442	3.2145199
hsa04810 Regulation of actin cytoskeleton	0.59038668	1.09981997	0.76503832
hsa04910 Insulin signaling pathway	1.09404656	0.1352316	0.18573026
hsa04911 Insulin secretion	0.28489626	1.05343614	0.65354423
hsa04912 GnRH signaling pathway	0.4160026	0.69026265	0.78294821
hsa04913 Ovarian steroidogenesis	0.28666618	0.75842459	1.55025317
hsa04914 Progesterone-mediated oocyte maturation	0.28263629	1.03566082	0.30727917
hsa04915 Estrogen signaling pathway	0.56307329	1.20537507	0.06773798
hsa04916 Melanogenesis	0.36194062	0.3848011	0.85725599
hsa04917 Prolactin signaling pathway	0.82432591	1.20476898	0.60457038
hsa04918 Thyroid hormone synthesis	0.56704944	0.5598669	0.28489626
hsa04919 Thyroid hormone signaling pathway	0.84592124	1.05343614	1.04486087
hsa04920 Adipocytokine signaling pathway	1.43121608	0.56704944	1.08501418
hsa04921 Oxytocin signaling pathway	0.28428077	1.56271724	0.78294821
hsa04922 Glucagon signaling pathway	0.54570427	0.37894248	0.34331287
hsa04960 Aldosterone-regulated sodium reabsorption	1.31857664	0.88476264	0.88476264
hsa04961 Endocrine and other factor-regulated calciun	0.30530343	0.78606864	0.30530343
hsa04962 Vasopressin-regulated water reabsorption	0.13116847	0.45408152	0.04760122
hsa04964 Proximal tubule bicarbonate reclamation	0.54570427	0.31428034	0.35364118
hsa04966 Collecting duct acid secretion	0.211747	0.65928509	0.12177361
hsa04970 Salivary secretion	0.42747802	0.44019414	0.43052127
hsa04971 Gastric acid secretion	0.5394339	0.83485935	0.58187188
hsa04972 Pancreatic secretion	0.59038668	0.6552596	0.53485508
hsa04973 Carbohydrate digestion and absorption	0.87162763	0.45408152	0.04760122
hsa04974 Protein digestion and absorption	0.64253303	1.30206728	0.99575064
hsa04975 Fat digestion and absorption	1.80907079	0.21775761	0.35364118
hsa04976 Bile secretion	0.56704944	1.20537507	0.83098243
hsa04977 Vitamin digestion and absorption	1.31857664	0.30610017	0.96758561
hsa04978 Mineral absorption	0.28428077	0.4160026	0.28263629
hsa00010 Glycolysis / Gluconeogenesis	0.36194062	0.1346108	0.45408152
hsa00020 Citrate cycle (TCA cycle)	0.19464349	0.27876652	0.28428077
hsa00030 Pentose phosphate pathway	0.45408152	1.07781407	0.82766809
hsa00040 Pentose and glucuronate interconversions	0.37387207	0.54287035	1.68440823
hsa00051 Fructose and mannose metabolism	0.42414457	0.26993475	0.27094648
hsa00052 Galactose metabolism	0.44053573	0.27876652	0.49755095
hsa00053 Ascorbate and aldarate metabolism	0.46924688	0.29100893	1.64706179

hsa00061 Fatty acid biosynthesis	2.964536	0.42695714	0.24837149
hsa00062 Fatty acid elongation	0.22553797	0.30530343	0.1346108
hsa00071 Fatty acid degradation	2.26999488	0.20452893	0.18047004
hsa00072 Synthesis and degradation of ketone bodies	0.37988252	0.46800068	0.29100893
hsa00100 Steroid biosynthesis	1.04486087	1.28155332	0.40123762
hsa00120 Primary bile acid biosynthesis	1.10808662	0.36194062	0.19890669
hsa00130 Ubiquinone and other terpenoid-quinone bio:	0.84696662	0.45408152	0.64253303
hsa00140 Steroid hormone biosynthesis	0.43585987	0.15738486	1.30206728
hsa00190 Oxidative phosphorylation	0.13534378	0.28489626	0.00549542
hsa00230 Purine metabolism	0.13551339	0.30727917	0.63611076
hsa00232 Caffeine metabolism	0.53800258	0.64253303	0.98704607
hsa00240 Pyrimidine metabolism	0.33836256	0.20452893	0.22337659
hsa00250 Alanine, aspartate and glutamate metabolisr	0.70771226	0.24837149	0.69026265
hsa00260 Glycine, serine and threonine metabolism	0.14308613	0.22288598	0.20093494
hsa00270 Cysteine and methionine metabolism	0.88476264	0.20452893	0.32160523
hsa00280 Valine, leucine and isoleucine degradation	0.30530343	0.19464349	0.04385493
hsa00290 Valine, leucine and isoleucine biosynthesis	0.59038668	0.69026265	0.45408152
hsa00300 Lysine biosynthesis	0.75184517	0.83877231	0.62315874
hsa00310 Lysine degradation	0.28263629	0.1798849	0.27876652
hsa00330 Arginine and proline metabolism	0.28666618	0.18573026	0.68827865
hsa00340 Histidine metabolism	0.95348518	0.31428034	0.65354423
hsa00350 Tyrosine metabolism	0.70771226	0.24837149	0.69026265
hsa00360 Phenylalanine metabolism	0.67219723	0.36194062	1.20699442
hsa00380 Tryptophan metabolism	0.96868685	0.22288598	0.36194062
hsa00400 Phenylalanine, tyrosine and tryptophan bios	1.20537507	0.64253303	0.98704607
hsa00410 beta-Alanine metabolism	0.43420076	0.27356757	0.78294821
hsa00430 Taurine and hypotaurine metabolism	0.36194062	0.45408152	0.28056609
hsa00450 Selenocompound metabolism	0.29100893	0.36194062	0.45408152
hsa00460 Cyanoamino acid metabolism	0.45408152	0.56294305	0.34957002
hsa00471 D-Glutamine and D-glutamate metabolism	0.59038668	0.69026265	1.08501418
hsa00472 D-Arginine and D-ornithine metabolism	0.88476264	0.98894236	0.78294821
hsa00480 Glutathione metabolism	0.28428077	0.18261675	0.03666085
hsa00500 Starch and sucrose metabolism	0.72726789	0.16436535	1.08501418
hsa00510 N-Glycan biosynthesis	0.82766809	0.18889409	0.45408152
hsa00511 Other glycan degradation	1.08501418	0.35714788	0.19172148
hsa00512 Mucin type O-Glycan biosynthesis	1.14845944	0.60457038	1.43192959
hsa00514 Other types of O-glycan biosynthesis	0.43420076	0.27356757	0.78294821
hsa00520 Amino sugar and nucleotide sugar metabolis	0.83099427	0.19172148	0.29754233
hsa00524 Butirosin and neomycin biosynthesis	1.20537507	0.64253303	0.42012315
hsa00531 Glycosaminoglycan degradation	0.62770512	0.34719976	0.18261675
hsa00532 Glycosaminoglycan biosynthesis - chondroiti	0.60457038	0.78512292	0.40123762
hsa00533 Glycosaminoglycan biosynthesis - keratan su	0.72531309	0.8850649	0.50629835
hsa00534 Glycosaminoglycan biosynthesis - heparan s	2.74847876	1.19069333	0.33836256
hsa00561 Glycerolipid metabolism	0.98704607	0.15462351	0.55697608
hsa00562 Inositol phosphate metabolism	0.56704944	0.85891916	0.42608461
hsa00563 Glycosylphosphatidylinositol(GPI)-anchor bio	0.22553797	0.30530343	0.1346108
hsa00564 Glycerophospholipid metabolism	1.08501418	0.23360708	0.54287035
hsa00565 Ether lipid metabolism	0.87162763	0.20093494	0.31428034
hsa00590 Arachidonic acid metabolism	0.22738515	0.14479506	0.74617128
hsa00591 Linoleic acid metabolism	0.45408152	0.28263629	0.52086844
hsa00592 alpha-Linolenic acid metabolism	0.51172707	0.30530343	0.60405584
hsa00600 Sphingolipid metabolism	0.84592124	0.19464349	0.04385493
hsa00601 Glycosphingolipid biosynthesis - lacto and ne	1.28155332	0.29539896	0.31428034
hsa00603 Glycosphingolipid biosynthesis - globo series	1.87654726	2.42901562	0.23360708
hsa00604 Glycosphingolipid biosynthesis - ganglio seri	1.20537507	0.8850649	0.50629835
hsa00620 Pyruvate metabolism	0.34468011	0.22288598	0.36194062
hsa00630 Glyoxylate and dicarboxylate metabolism	0.45408152	0.28489626	0.11580111
hsa00640 Propanoate metabolism	0.18573026	0.26993475	0.45408152
hsa00650 Butanoate metabolism	0.211747	0.29100893	0.12177361
hsa00670 One carbon pool by folate	0.27110033	0.33747584	0.40123762
hsa00730 Thiamine metabolism	0.59038668	0.69026265	0.45408152
hsa00740 Riboflavin metabolism	0.59038668	0.69026265	1.08501418
hsa00750 Vitamin B6 metabolism	0.48561659	0.5958386	1.51492444
hsa00760 Nicotinate and nicotinamide metabolism	0.82404684	1.07781407	0.52086844
hsa00770 Pantothenate and CoA biosynthesis	1.08501418	0.35714788	0.19172148
hsa00780 Biotin metabolism	0.65354423	0.75842459	1.20699442
hsa00785 Lipoic acid metabolism	0.65354423	0.75842459	0.53353389
hsa00790 Folate biosynthesis	0.31596355	0.4106878	0.23360708
hsa00830 Retinol metabolism	0.06634004	0.13551339	1.43192959
hsa00860 Porphyrin and chlorophyll metabolism	0.13534378	0.21206425	1.43121608
hsa00900 Terpenoid backbone biosynthesis	0.24931982	0.31596355	0.15462351
hsa00910 Nitrogen metabolism	0.67219723	0.36194062	0.19890669

hsa00920	Sulfur metabolism	0.88476264	0.46800068	0.67509174
hsa00980	Metabolism of xenobiotics by cytochrome P4	0.32608645	0.118757	1.02696491
hsa00982	Drug metabolism - cytochrome P450	0.20219592	0.1324597	0.87162763
hsa00983	Drug metabolism - other enzymes	0.30565481	0.19776214	1.30206728
hsa01040	Biosynthesis of unsaturated fatty acids	0.24263714	0.31428034	0.14482444
hsa01100	Metabolic pathways	1.80907079	0.04760122	0.64634045
hsa01200	Carbon metabolism	0.30727917	0.33747584	0.54287035
hsa01210	2-Oxocarboxylic acid metabolism	0.67219723	0.36194062	0.45408152
hsa01212	Fatty acid metabolism	1.55025317	0.19172148	0.04185459
hsa01220	Degradation of aromatic compounds	0.65354423	0.75842459	0.53353389
hsa01230	Biosynthesis of amino acids	0.5394339	0.53800258	0.58187188

Supplementary Table S2 - List of ChIP-Seq datasets used in the study (MCF7 cells)

Biosample_name	Target	Experiment	Accession
MCF-7	Control	Data will be provided through GEO upon publication	
MCF-7	NIPBL	Data will be provided through GEO upon publication	
MCF-7	MED1	Data will be provided through GEO upon publication	
MCF-7	SMC1A	Data will be provided through GEO upon publication	
MCF-7	Era	Data will be provided through GEO upon publication	
MCF-7	CEBPB	ENCSR000BSR	ENCFF000QLL
MCF-7	CEBPB	ENCSR000BSR	ENCFF000QLO
MCF-7	Control	ENCSR000EWW	ENCFF000VHM
MCF-7	Control	ENCSR000EWW	ENCFF000VHL
MCF-7	Control	ENCSR000DWI	ENCFF001HUM
MCF-7	Control	ENCSR000DMW	ENCFF000SAZ
MCF-7	Control	ENCSR000AHE	ENCFF000QQJ
MCF-7	Control	ENCSR000AHE	ENCFF000QQG
MCF-7	Control	ENCSR000AHE	ENCFF000QQI
MCF-7	Control	ENCSR000AHE	ENCFF000QQK
MCF-7	CTCF	ENCSR000DWH	ENCFF001HUF
MCF-7	CTCF	ENCSR000DWH	ENCFF001HUG
MCF-7	CTCF	ENCSR000DMV	ENCFF000RZX
MCF-7	CTCF	ENCSR000DMV	ENCFF000RZY
MCF-7	CTCF	ENCSR000DMS	ENCFF000SAB
MCF-7	CTCF	ENCSR000DMS	ENCFF000SAD
MCF-7	CTCF	ENCSR000DMR	ENCFF000SAY
MCF-7	CTCF	ENCSR000DMR	ENCFF000SAX
MCF-7	CTCF	ENCSR000DMO	ENCFF000SAL
MCF-7	CTCF	ENCSR000DMO	ENCFF000SAK
MCF-7	CTCF	ENCSR000DML	ENCFF000SAP
MCF-7	CTCF	ENCSR000DML	ENCFF000SAO
MCF-7	CTCF	ENCSR000AHD	ENCFF000QLW
MCF-7	CTCF	ENCSR000AHD	ENCFF000QLT
MCF-7	EGR1	ENCSR000BUX	ENCFF000QMF
MCF-7	EGR1	ENCSR000BUX	ENCFF000QMC
MCF-7	ELF1	ENCSR000BSS	ENCFF000QMJ
MCF-7	ELF1	ENCSR000BSS	ENCFF000QMK
MCF-7	EP300	ENCSR000BTR	ENCFF000QPK
MCF-7	EP300	ENCSR000BTR	ENCFF000QPP
MCF-7	FOSL2	ENCSR000BUI	ENCFF000QMU
MCF-7	FOSL2	ENCSR000BUI	ENCFF000QMS
MCF-7	FOXM1	ENCSR000BUJ	ENCFF000QMZ
MCF-7	FOXM1	ENCSR000BUJ	ENCFF000QNC
MCF-7	GABPA	ENCSR000BUK	ENCFF000QNK
MCF-7	GABPA	ENCSR000BUK	ENCFF000QNJ
MCF-7	GATA3	ENCSR000EWV	ENCFF000ZKL
MCF-7	GATA3	ENCSR000EWV	ENCFF000ZKJ
MCF-7	GATA3	ENCSR000EWS	ENCFF000ZKV
MCF-7	GATA3	ENCSR000EWS	ENCFF000ZKT
MCF-7	GATA3	ENCSR000EWS	ENCFF000ZLE
MCF-7	GATA3	ENCSR000BST	ENCFF000QNS
MCF-7	GATA3	ENCSR000BST	ENCFF000QNP
MCF-7	H3K27ac	ENCSR000EWR	ENCFF000VHD

MCF-7	H3K27ac	ENCSR000EWR	ENCFF000VHC
MCF-7	H3K27me3	ENCSR000EWP	ENCFF000VFU
MCF-7	H3K27me3	ENCSR000EWP	ENCFF000VFY
MCF-7	H3K27me3	ENCSR000EWP	ENCFF000VFW
MCF-7	H3K36me3	ENCSR000EWO	ENCFF000VGE
MCF-7	H3K36me3	ENCSR000EWO	ENCFF000VGG
MCF-7	H3K36me3	ENCSR000EWO	ENCFF000VGI
MCF-7	H3K4me3	ENCSR000DWJ	ENCFF001FYK
MCF-7	H3K4me3	ENCSR000DWJ	ENCFF001FYE
MCF-7	H3K9me3	ENCSR000EWQ	ENCFF000VFG
MCF-7	H3K9me3	ENCSR000EWQ	ENCFF000VFJ
MCF-7	H3K9me3	ENCSR000EWQ	ENCFF000VFE
MCF-7	HA-E2F1	ENCSR000EWX	ENCFF000ZLD
MCF-7	HA-E2F1	ENCSR000EWX	ENCFF000ZLV
MCF-7	HDAC2	ENCSR000BTP	ENCFF000QNY
MCF-7	HDAC2	ENCSR000BTP	ENCFF000QOA
MCF-7	JUND	ENCSR000BSU	ENCFF000QOD
MCF-7	JUND	ENCSR000BSU	ENCFF000QOB
MCF-7	MAX	ENCSR000BUL	ENCFF000QON
MCF-7	MAX	ENCSR000BUL	ENCFF000QOS
MCF-7	MYC	ENCSR000DMQ	ENCFF000RZT
MCF-7	MYC	ENCSR000DMQ	ENCFF000RZV
MCF-7	MYC	ENCSR000DMP	ENCFF000RYO
MCF-7	MYC	ENCSR000DMP	ENCFF000RYP
MCF-7	MYC	ENCSR000DMM	ENCFF000RZE
MCF-7	MYC	ENCSR000DMM	ENCFF000RZC
MCF-7	MYC	ENCSR000DMJ	ENCFF000RZL
MCF-7	MYC	ENCSR000DMJ	ENCFF000RZJ
MCF-7	NR2F2	ENCSR000BUY	ENCFF000QOY
MCF-7	NR2F2	ENCSR000BUY	ENCFF000QOV
MCF-7	PML	ENCSR000BUZ	ENCFF000QPW
MCF-7	PML	ENCSR000BUZ	ENCFF000QPV
MCF-7	POLR2A	ENCSR000DMT	ENCFF000SBK
MCF-7	POLR2A	ENCSR000DMT	ENCFF000SBI
MCF-7	POLR2A	ENCSR000DMN	ENCFF000SBP
MCF-7	POLR2A	ENCSR000DMN	ENCFF000SBO
MCF-7	POLR2A	ENCSR000DMK	ENCFF000SBV
MCF-7	POLR2A	ENCSR000DMK	ENCFF000SBY
MCF-7	RAD21	ENCSR000BTQ	ENCFF000QPX
MCF-7	RAD21	ENCSR000BTQ	ENCFF000QQA
MCF-7	REST	ENCSR000BSP	ENCFF000QPH
MCF-7	REST	ENCSR000BSP	ENCFF000QPE
MCF-7	SIN3A	ENCSR000BUM	ENCFF000QQX
MCF-7	SIN3A	ENCSR000BUM	ENCFF000QQU
MCF-7	SRF	ENCSR000BVA	ENCFF000QRE
MCF-7	SRF	ENCSR000BVA	ENCFF000QRH
MCF-7	TAF1	ENCSR000AHF	ENCFF000QRS
MCF-7	TAF1	ENCSR000AHF	ENCFF000QRL
MCF-7	TCF12	ENCSR000BUN	ENCFF000QRV
MCF-7	TCF12	ENCSR000BUN	ENCFF000QRU
MCF-7	TCF7L2	ENCSR000EWT	ENCFF000ZLK
MCF-7	TCF7L2	ENCSR000EWT	ENCFF000ZLM

MCF-7	TEAD4	ENCSR000BUO	ENCFF000QSI
MCF-7	TEAD4	ENCSR000BUO	ENCFF000QSB
MCF-7	ZNF217	ENCSR000EWU	ENCFF000ZLW
MCF-7	ZNF217	ENCSR000EWU	ENCFF000ZLT
MCF-7	FOXA1	SRP044607/SRX747793	SRR1635461
MCF-7	FOXA1	SRP044607/SRX747794	SRR1635462
MCF-7	Control	SRP044607	SRR1635437
MCF-7	Control	SRP044607	SRR1635438
MCF-7	H3K4me1	SRX153145	SRR507786
MCF-7	H3K4me1	SRX153146	SRR507787
MCF-7	H3K4me1	SRX153147	SRR507788

Supplementary Table S2 - List of ChIP-Seq datasets used in the study (HEPG2 cells)

Biosample_name	Target	Experiment	Accession
HEPG2	Control	Data will be provided through GEO upon publication	
HEPG2	NIPBL	Data will be provided through GEO upon publication	
HEPG2	MED1	Data will be provided through GEO upon publication	
HEPG2	SMC1A	Data will be provided through GEO upon publication	
HepG2	ARID3A	ENCSR000EDP	ENCFF000XOU
HepG2	ARID3A	ENCSR000EDP	ENCFF000XOS
HepG2	ATF3	ENCSR000BKE	ENCFF000PFR
HepG2	ATF3	ENCSR000BKE	ENCFF000PFQ
HepG2	BHLHE40	ENCSR000EDT	ENCFF000XPC
HepG2	BHLHE40	ENCSR000EDT	ENCFF000XPA
HepG2	BHLHE40	ENCSR000BID	ENCFF000PFY
HepG2	BHLHE40	ENCSR000BID	ENCFF000PGB
HepG2	BRCA1	ENCSR000EDY	ENCFF000XPS
HepG2	BRCA1	ENCSR000EDY	ENCFF000XPL
HepG2	CEBPB	ENCSR000EEX	ENCFF000XPT
HepG2	CEBPB	ENCSR000EEX	ENCFF000XPR
HepG2	CEBPB	ENCSR000EEE	ENCFF000XQN
HepG2	CEBPB	ENCSR000EEE	ENCFF000XQM
HepG2	CEBPB	ENCSR000BQI	ENCFF000PGI
HepG2	CEBPB	ENCSR000BQI	ENCFF000PGG
HepG2	CEBPD	ENCSR000BQJ	ENCFF000PGK
HepG2	CEBPD	ENCSR000BQJ	ENCFF000PGQ
HepG2	CEBPZ	ENCSR000EDO	ENCFF000XPZ
HepG2	CEBPZ	ENCSR000EDO	ENCFF000XQB
HepG2	CHD2	ENCSR000EED	ENCFF000XQQ
HepG2	CHD2	ENCSR000EED	ENCFF000XQP
HepG2	Control	ENCSR000DUC	ENCFF001HOD
HepG2	Control	ENCSR000EES	ENCFF000XQR
HepG2	Control	ENCSR000EES	ENCFF000XQS
HepG2	Control	ENCSR000EER	ENCFF000XRI
HepG2	Control	ENCSR000EER	ENCFF000XRH
HepG2	Control	ENCSR000EVT	ENCFF000XTE
HepG2	Control	ENCSR000AME	ENCFF000BDZ
HepG2	Control	ENCSR000AME	ENCFF000BDT
HepG2	Control	ENCSR000DLT	ENCFF000RUP
HepG2	Control	ENCSR000EZR	ENCFF000XTB
HepG2	Control	ENCSR000EZR	ENCFF000XSV
HepG2	Control	ENCSR000EZR	ENCFF000XSY
HepG2	Control	ENCSR000BLN	ENCFF000POH
HepG2	Control	ENCSR000BLN	ENCFF000POC
HepG2	Control	ENCSR000BPH	ENCFF000PPI
HepG2	Control	ENCSR000BPH	ENCFF000PPF
HepG2	Control	ENCSR000BPH	ENCFF000PPC
HepG2	Control	ENCSR000BLH	ENCFF000POO
HepG2	Control	ENCSR000BLH	ENCFF000POQ
HepG2	Control	ENCSR000BLG	ENCFF000POU
HepG2	Control	ENCSR000BLG	ENCFF000POV
HepG2	CREB1	ENCSR000BVL	ENCFF000PGX
HepG2	CREB1	ENCSR000BVL	ENCFF000PGZ
HepG2	CTCF	ENCSR000DUG	ENCFF001HNU
HepG2	CTCF	ENCSR000DUG	ENCFF001HNT
HepG2	CTCF	ENCSR000AMA	ENCFF000BEI
HepG2	CTCF	ENCSR000AMA	ENCFF000BED
HepG2	CTCF	ENCSR000BIE	ENCFF000PHG

HepG2	CTCF	ENCSR000BIE	ENCFF000PHE
HepG2	CTCF	ENCSR000DLS	ENCFF000RUI
HepG2	CTCF	ENCSR000DLS	ENCFF000RUJ
HepG2	ELF1	ENCSR000BMZ	ENCFF000PHP
HepG2	ELF1	ENCSR000BMZ	ENCFF000PHM
HepG2	EP300	ENCSR000EDV	ENCFF000XVS
HepG2	EP300	ENCSR000EDV	ENCFF000XVT
HepG2	EP300	ENCSR000BLW	ENCFF000PMZ
HepG2	EP300	ENCSR000BLW	ENCFF000PNA
HepG2	ESRRA	ENCSR000EEW	ENCFF000XRN
HepG2	ESRRA	ENCSR000EEW	ENCFF000XRO
HepG2	EZH2	ENCSR000ARI	ENCFF000BEL
HepG2	EZH2	ENCSR000ARI	ENCFF000BEQ
HepG2	FOSL2	ENCSR000BHP	ENCFF000PHU
HepG2	FOSL2	ENCSR000BHP	ENCFF000PIE
HepG2	FOXA1	ENCSR000BMO	ENCFF000PIB
HepG2	FOXA1	ENCSR000BMO	ENCFF000PHY
HepG2	FOXA1	ENCSR000BLE	ENCFF000PIM
HepG2	FOXA1	ENCSR000BLE	ENCFF000PIJ
HepG2	FOXA2	ENCSR000BNI	ENCFF000PIX
HepG2	FOXA2	ENCSR000BNI	ENCFF000PIT
HepG2	GABPA	ENCSR000BJK	ENCFF000PJF
HepG2	GABPA	ENCSR000BJK	ENCFF000PJC
HepG2	goat-IgG-control	ENCSR000EDR	ENCFF000XSE
HepG2	goat-IgG-control	ENCSR000EDR	ENCFF000XSG
HepG2	H2AFZ	ENCSR000AOK	ENCFF000BEP
HepG2	H2AFZ	ENCSR000AOK	ENCFF000BES
HepG2	H3K27ac	ENCSR000AMO	ENCFF000BFD
HepG2	H3K27ac	ENCSR000AMO	ENCFF000BFH
HepG2	H3K27me3	ENCSR000DUE	ENCFF001FLT
HepG2	H3K27me3	ENCSR000DUE	ENCFF001FLQ
HepG2	H3K27me3	ENCSR000AOL	ENCFF000BFR
HepG2	H3K27me3	ENCSR000AOL	ENCFF000BFU
HepG2	H3K36me3	ENCSR000DUD	ENCFF001FMA
HepG2	H3K36me3	ENCSR000DUD	ENCFF001FMC
HepG2	H3K36me3	ENCSR000AMB	ENCFF000BFZ
HepG2	H3K36me3	ENCSR000AMB	ENCFF000BFT
HepG2	H3K4me1	ENCSR000APV	ENCFF000BEY
HepG2	H3K4me1	ENCSR000APV	ENCFF000BEX
HepG2	H3K4me2	ENCSR000AMC	ENCFF000BGD
HepG2	H3K4me2	ENCSR000AMC	ENCFF000BGA
HepG2	H3K4me3	ENCSR000DUF	ENCFF001FMH
HepG2	H3K4me3	ENCSR000DUF	ENCFF001FMG
HepG2	H3K4me3	ENCSR000AMP	ENCFF000BGF
HepG2	H3K4me3	ENCSR000AMP	ENCFF000BGE
HepG2	H3K79me2	ENCSR000AOM	ENCFF000BGR
HepG2	H3K79me2	ENCSR000AOM	ENCFF000BGW
HepG2	H3K9ac	ENCSR000AMD	ENCFF000BGQ
HepG2	H3K9ac	ENCSR000AMD	ENCFF000BGS
HepG2	H3K9me3	ENCSR000ATD	ENCFF000BFK
HepG2	H3K9me3	ENCSR000ATD	ENCFF000BFI
HepG2	H4K20me1	ENCSR000AMQ	ENCFF000BGX
HepG2	H4K20me1	ENCSR000AMQ	ENCFF000BHC
HepG2	HDAC2	ENCSR000BMC	ENCFF000PJJ
HepG2	HDAC2	ENCSR000BMC	ENCFF000PJD
HepG2	HNF4A	ENCSR000EEU	ENCFF000XRT
HepG2	HNF4A	ENCSR000EEU	ENCFF000XSM

HepG2	HNF4A	ENCSR000BLF	ENCFF000PJT
HepG2	HNF4A	ENCSR000BLF	ENCFF000PJX
HepG2	HNF4G	ENCSR000BNJ	ENCFF000PKH
HepG2	HNF4G	ENCSR000BNJ	ENCFF000PKJ
HepG2	HSF1	ENCSR000EET	ENCFF000XSL
HepG2	HSF1	ENCSR000EET	ENCFF000XSK
HepG2	IRF3	ENCSR000EEJ	ENCFF000XTN
HepG2	IRF3	ENCSR000EEJ	ENCFF000XTO
HepG2	JUN	ENCSR000EEK	ENCFF000XQU
HepG2	JUN	ENCSR000EEK	ENCFF000XQT
HepG2	JUND	ENCSR000EEI	ENCFF000XTR
HepG2	JUND	ENCSR000EEI	ENCFF000XTQ
HepG2	JUND	ENCSR000BGK	ENCFF000PKR
HepG2	JUND	ENCSR000BGK	ENCFF000PKK
HepG2	MAFF	ENCSR000EEC	ENCFF000XTZ
HepG2	MAFF	ENCSR000EEC	ENCFF000XUA
HepG2	MAFK	ENCSR000EEB	ENCFF000XUK
HepG2	MAFK	ENCSR000EEB	ENCFF000XUL
HepG2	MAFK	ENCSR000EDZ	ENCFF000XUU
HepG2	MAFK	ENCSR000EDZ	ENCFF000XVK
HepG2	MAX	ENCSR000EDS	ENCFF000XUH
HepG2	MAX	ENCSR000EDS	ENCFF000XUF
HepG2	MAX	ENCSR000BTM	ENCFF000PKZ
HepG2	MAX	ENCSR000BTM	ENCFF000PKX
HepG2	MAZ	ENCSR000EDN	ENCFF000XUP
HepG2	MAZ	ENCSR000EDN	ENCFF000XUN
HepG2	MBD4	ENCSR000BQW	ENCFF000PLF
HepG2	MBD4	ENCSR000BQW	ENCFF000PLH
HepG2	MXI1	ENCSR000EDU	ENCFF000XVA
HepG2	MXI1	ENCSR000EDU	ENCFF000XUX
HepG2	MXI1	ENCSR000EDU	ENCFF000XVC
HepG2	MYBL2	ENCSR000BRO	ENCFF000PLP
HepG2	MYBL2	ENCSR000BRO	ENCFF000PLM
HepG2	MYC	ENCSR000DLR	ENCFF000RTV
HepG2	MYC	ENCSR000DLR	ENCFF000RTX
HepG2	MYC	ENCSR000DLR	ENCFF000RTY
HepG2	NFIC	ENCSR000BQX	ENCFF000PMB
HepG2	NFIC	ENCSR000BQX	ENCFF000PLW
HepG2	NR2C2	ENCSR000EVS	ENCFF000XZK
HepG2	NR2C2	ENCSR000EVS	ENCFF000XZJ
HepG2	NR2F2	ENCSR000BVM	ENCFF000PMC
HepG2	NR2F2	ENCSR000BVM	ENCFF000PMF
HepG2	NR3C1	ENCSR000EEV	ENCFF000XRV
HepG2	NR3C1	ENCSR000EEV	ENCFF000XRU
HepG2	NRF1	ENCSR000EEH	ENCFF000XVP
HepG2	NRF1	ENCSR000EEH	ENCFF000XVR
HepG2	POLR2A	ENCSR000EEM	ENCFF000XWJ
HepG2	POLR2A	ENCSR000EEM	ENCFF000XWR
HepG2	POLR2A	ENCSR000EEP	ENCFF000XWF
HepG2	POLR2A	ENCSR000EEP	ENCFF000XWI
HepG2	POLR2A	ENCSR000EZQ	ENCFF000XWW
HepG2	POLR2A	ENCSR000EZQ	ENCFF000XWT
HepG2	POLR2A	ENCSR000EZQ	ENCFF000XWV
HepG2	POLR2A	ENCSR000DLQ	ENCFF000RUT
HepG2	POLR2A	ENCSR000DLQ	ENCFF000RUR
HepG2	POLR2A	ENCSR000DLQ	ENCFF000RUY
HepG2	POLR2A	ENCSR000BJM	ENCFF000PNT

HepG2	POLR2A	ENCSR000BJM	ENCFF000PNM
HepG2	POLR2AphosphoS2	ENCSR000EDX	ENCFF000XXB
HepG2	POLR2AphosphoS2	ENCSR000EDX	ENCFF000XXC
HepG2	POLR2AphosphoS5	ENCSR000BPI	ENCFF000PNI
HepG2	POLR2AphosphoS5	ENCSR000BPI	ENCFF000PNL
HepG2	PPARGC1A	ENCSR000EEQ	ENCFF000XVQ
HepG2	PPARGC1A	ENCSR000EEQ	ENCFF000XWG
HepG2	rabbit-IgG-control	ENCSR000EEN	ENCFF000XTF
HepG2	RAD21	ENCSR000EEG	ENCFF000XXK
HepG2	RAD21	ENCSR000EEG	ENCFF000XXL
HepG2	RAD21	ENCSR000BLS	ENCFF000PNY
HepG2	RAD21	ENCSR000BLS	ENCFF000PNW
HepG2	RCOR1	ENCSR000EDQ	ENCFF000XQY
HepG2	RCOR1	ENCSR000EDQ	ENCFF000XQW
HepG2	REST	ENCSR000BJL	ENCFF000PMN
HepG2	REST	ENCSR000BJL	ENCFF000PMK
HepG2	REST	ENCSR000BOT	ENCFF000PMU
HepG2	REST	ENCSR000BOT	ENCFF000PMV
HepG2	RFX5	ENCSR000EEA	ENCFF000XXZ
HepG2	RFX5	ENCSR000EEA	ENCFF000XXX
HepG2	RXRA	ENCSR000BHU	ENCFF000PPQ
HepG2	RXRA	ENCSR000BHU	ENCFF000PPM
HepG2	SIN3A	ENCSR000BGL	ENCFF000PPW
HepG2	SIN3A	ENCSR000BGL	ENCFF000PQC
HepG2	SMC3	ENCSR000EDW	ENCFF000XXY
HepG2	SMC3	ENCSR000EDW	ENCFF000XYC
HepG2	SP1	ENCSR000BJX	ENCFF000PQJ
HepG2	SP1	ENCSR000BJX	ENCFF000PQE
HepG2	SP2	ENCSR000BOU	ENCFF000PQL
HepG2	SP2	ENCSR000BOU	ENCFF000PQP
HepG2	SREBF1	ENCSR000EEO	ENCFF000XYE
HepG2	SREBF1	ENCSR000EEO	ENCFF000XYA
HepG2	SREBF1	ENCSR000EEO	ENCFF000XYB
HepG2	SREBF1	ENCSR000EZP	ENCFF000XYK
HepG2	SREBF1	ENCSR000EZP	ENCFF000XYD
HepG2	SREBF1	ENCSR000EZP	ENCFF000XYL
HepG2	SREBF2	ENCSR000EZO	ENCFF000XYM
HepG2	SREBF2	ENCSR000EZO	ENCFF000XYN
HepG2	SREBF2	ENCSR000EZO	ENCFF000XYS
HepG2	SRF	ENCSR000BLV	ENCFF000PQW
HepG2	SRF	ENCSR000BLV	ENCFF000PQU
HepG2	TAF1	ENCSR000BJN	ENCFF000PRD
HepG2	TAF1	ENCSR000BJN	ENCFF000PRB
HepG2	TBP	ENCSR000EEL	ENCFF000XZI
HepG2	TBP	ENCSR000EEL	ENCFF000XZL
HepG2	TCF12	ENCSR000BJG	ENCFF000PRJ
HepG2	TCF12	ENCSR000BJG	ENCFF000PRL
HepG2	TCF7L2	ENCSR000EVQ	ENCFF000XYX
HepG2	TCF7L2	ENCSR000EVQ	ENCFF000XYV
HepG2	TEAD4	ENCSR000BRP	ENCFF000PRQ
HepG2	TEAD4	ENCSR000BRP	ENCFF000PRU
HepG2	USF1	ENCSR000BGM	ENCFF000PSH
HepG2	USF1	ENCSR000BGM	ENCFF000PSA
HepG2	USF2	ENCSR000EEF	ENCFF000XZT
HepG2	USF2	ENCSR000EEF	ENCFF000XZV
HepG2	YY1	ENCSR000BNT	ENCFF000PSE
HepG2	YY1	ENCSR000BNT	ENCFF000PSD

HepG2	ZBTB33	ENCSR000BNA	ENCFF000PSY
HepG2	ZBTB33	ENCSR000BNA	ENCFF000PST
HepG2	ZBTB33	ENCSR000BHR	ENCFF000PSP
HepG2	ZBTB33	ENCSR000BHR	ENCFF000PSW
HepG2	ZBTB7A	ENCSR000BQA	ENCFF000PTI
HepG2	ZBTB7A	ENCSR000BQA	ENCFF000PTF
HepG2	ZEB1	ENCSR000BVN	ENCFF000PTQ
HepG2	ZEB1	ENCSR000BVN	ENCFF000PTO
HepG2	ZNF274	ENCSR000EVR	ENCFF000XZW
HepG2	ZNF274	ENCSR000EVR	ENCFF000XZX

Supplementary Table S2 - List of ChIP-Seq datasets used in the study (A549 cells)

Biosample_name	Target	Experiment	Accession
A549	Control	Data will be provided through GEO upon publication	
A549	NIPBL	Data will be provided through GEO upon publication	
A549	MED1	Data will be provided through GEO upon publication	
A549	SMC1A	Data will be provided through GEO upon publication	
A549	ATF3	ENCSR000BPS	ENCFF000MWR
A549	ATF3	ENCSR000BPS	ENCFF000MWV
A549	BCL3	ENCSR000BQH	ENCFF000MXC
A549	BCL3	ENCSR000BQH	ENCFF000MXD
A549	BHLHE40	ENCSR000DYJ	ENCFF000VOD
A549	CEBPB	ENCSR000DYI	ENCFF000VOL
A549	CEBPB	ENCSR000DYI	ENCFF000VON
A549	CEBPB	ENCSR000BUB	ENCFF000MXK
A549	CEBPB	ENCSR000BUB	ENCFF000MXH
A549	Control	ENCSR000DPE	ENCFF001GWE
A549	Control	ENCSR000DNB	ENCFF000RMI
A549	Control	ENCSR000BVQ	ENCFF000NGN
A549	Control	ENCSR000BVQ	ENCFF000NGK
A549	Control	ENCSR000BVQ	ENCFF000NGM
A549	Control	ENCSR000BPN	ENCFF000NGB
A549	Control	ENCSR000BPD	ENCFF000NGH
A549	Control	ENCSR000BNC	ENCFF000NFR
A549	Control	ENCSR000BKZ	ENCFF000NGC
A549	Control	ENCSR000ASS	ENCFF000AHV
A549	Control	ENCSR000ASS	ENCFF000AHM
A549	CREB1	ENCSR000BRC	ENCFF000MXT
A549	CREB1	ENCSR000BRA	ENCFF000MXO
A549	CTCF	ENCSR000DYD	ENCFF000VPF
A549	CTCF	ENCSR000DYD	ENCFF000VPD
A549	CTCF	ENCSR000DPF	ENCFF001GVU
A549	CTCF	ENCSR000DPF	ENCFF001GWB
A549	CTCF	ENCSR000DNA	ENCFF000RMB
A549	CTCF	ENCSR000DNA	ENCFF000RMC
A549	CTCF	ENCSR000BHW	ENCFF000MYJ
A549	CTCF	ENCSR000BHW	ENCFF000MYN
A549	CTCF	ENCSR000AUF	ENCFF000AHW
A549	CTCF	ENCSR000AUF	ENCFF000AHX
A549	E2F6	ENCSR000BTC	ENCFF000MYY
A549	E2F6	ENCSR000BTC	ENCFF000MYX
A549	ELF1	ENCSR000BPT	ENCFF000MZF
A549	ELF1	ENCSR000BPT	ENCFF000MZG
A549	EP300	ENCSR000BPW	ENCFF000NEG
A549	EP300	ENCSR000BPW	ENCFF000NEI
A549	ETS1	ENCSR000BPU	ENCFF000MZO
A549	ETS1	ENCSR000BPU	ENCFF000MZH
A549	FOSL2	ENCSR000BQO	ENCFF000MZW
A549	FOSL2	ENCSR000BQO	ENCFF000MZT
A549	FOXA1	ENCSR000BRD	ENCFF000NAI
A549	FOXA1	ENCSR000BRD	ENCFF000NAH
A549	FOXA2	ENCSR000BRE	ENCFF000NAQ
A549	FOXA2	ENCSR000BRE	ENCFF000NAU
A549	GABPA	ENCSR000BPY	ENCFF000NAY
A549	GABPA	ENCSR000BPY	ENCFF000NBA
A549	GATA3	ENCSR000BTI	ENCFF000NBH
A549	GATA3	ENCSR000BTI	ENCFF000NBM
A549	H2AFZ	ENCSR000AUH	ENCFF000AIK

A549	H2AFZ	ENCSR000AUH	ENCFF000AIJ
A549	H3K27ac	ENCSR000AUI	ENCFF000AKS
A549	H3K27ac	ENCSR000AUI	ENCFF000AKR
A549	H3K27me3	ENCSR000AUK	ENCFF000ALD
A549	H3K27me3	ENCSR000AUK	ENCFF000ALK
A549	H3K36me3	ENCSR000AUL	ENCFF000ALU
A549	H3K36me3	ENCSR000AUL	ENCFF000ALQ
A549	H3K4me1	ENCSR000AUM	ENCFF000AJC
A549	H3K4me1	ENCSR000AUM	ENCFF000AIW
A549	H3K4me2	ENCSR000AVI	ENCFF000AJI
A549	H3K4me2	ENCSR000AVI	ENCFF000AJH
A549	H3K4me3	ENCSR000DPD	ENCFF001EMB
A549	H3K4me3	ENCSR000DPD	ENCFF001EMC
A549	H3K4me3	ENCSR000ASH	ENCFF000AJT
A549	H3K4me3	ENCSR000ASH	ENCFF000AJX
A549	H3K79me2	ENCSR000ATP	ENCFF000AMB
A549	H3K79me2	ENCSR000ATP	ENCFF000AMD
A549	H3K9ac	ENCSR000ASV	ENCFF000AJY
A549	H3K9ac	ENCSR000ASV	ENCFF000AJZ
A549	H3K9me3	ENCSR000AUN	ENCFF000AKK
A549	H3K9me3	ENCSR000AUN	ENCFF000AKG
A549	H4K20me1	ENCSR000AUO	ENCFF000AMF
A549	H4K20me1	ENCSR000AUO	ENCFF000AME
A549	JUND	ENCSR000BRF	ENCFF000NDF
A549	JUND	ENCSR000BRF	ENCFF000NDH
A549	MAX	ENCSR000DYG	ENCFF000VPS
A549	MAX	ENCSR000DYG	ENCFF000VPU
A549	MAX	ENCSR000BTJ	ENCFF000NDP
A549	MAX	ENCSR000BTJ	ENCFF000NDT
A549	MYC	ENCSR000DYC	ENCFF000VOW
A549	MYC	ENCSR000DYC	ENCFF000VOU
A549	NR3C1	ENCSR000BJT	ENCFF000NDD
A549	NR3C1	ENCSR000BJT	ENCFF000NDA
A549	NR3C1	ENCSR000BIO	ENCFF000NCL
A549	NR3C1	ENCSR000BIO	ENCFF000NCO
A549	PBX3	ENCSR000BTN	ENCFF000NEY
A549	PBX3	ENCSR000BTN	ENCFF000NEP
A549	POLR2A	ENCSR000DMZ	ENCFF000RMM
A549	POLR2A	ENCSR000DMZ	ENCFF000RMO
A549	POLR2A	ENCSR000BHH	ENCFF000NEZ
A549	POLR2A	ENCSR000BHH	ENCFF000NFG
A549	POLR2AphosphoS2	ENCSR000DYF	ENCFF000VPZ
A549	POLR2AphosphoS2	ENCSR000DYF	ENCFF000VQB
A549	rabbit-IgG-control	ENCSR496AXR	ENCFF000VPI
A549	rabbit-IgG-control	ENCSR496AXR	ENCFF000VPK
A549	RAD21	ENCSR000DYE	ENCFF000VQI
A549	RAD21	ENCSR000DYE	ENCFF000VQK
A549	RAD21	ENCSR000BUC	ENCFF000NFN
A549	RAD21	ENCSR000BUC	ENCFF000NFL
A549	REST	ENCSR000BQP	ENCFF000NDY
A549	REST	ENCSR000BQP	ENCFF000NDV
A549	SIN3A	ENCSR000BRM	ENCFF000NGY
A549	SIN3A	ENCSR000BRM	ENCFF000NHA
A549	SIX5	ENCSR000BRL	ENCFF000NHG
A549	SIX5	ENCSR000BRL	ENCFF000NHI
A549	SP1	ENCSR000BPE	ENCFF000NHM
A549	SP1	ENCSR000BPE	ENCFF000NHP
A549	TAF1	ENCSR000BPF	ENCFF000NHV

A549	TAF1	ENCSR000BPF	ENCFF000NHY
A549	TCF12	ENCSR000BQQ	ENCFF000NIG
A549	TCF12	ENCSR000BQQ	ENCFF000NID
A549	TEAD4	ENCSR000BUD	ENCFF000NIP
A549	TEAD4	ENCSR000BUD	ENCFF000NIM
A549	USF1	ENCSR000BPV	ENCFF000NJL
A549	USF1	ENCSR000BPV	ENCFF000NJM
A549	USF1	ENCSR000BJB	ENCFF000NJA
A549	USF1	ENCSR000BJB	ENCFF000NJE
A549	YY1	ENCSR000BPM	ENCFF000NJU
A549	YY1	ENCSR000BPM	ENCFF000NJS
A549	ZBTB33	ENCSR000BPZ	ENCFF000NKG
A549	ZBTB33	ENCSR000BPZ	ENCFF000NKB

Supplementary Table S3 - List of shRNA plasmids and sequences used in the study

Targets	Reference Numbers	shRNA sequences
shCTRL-1	Addgene (#1864)	CCTAAGGTTAAGTCGCCCTCGCTCGAGCGAGGGCGACTTAACCTTAGG
shCTRL-2	SHC002 (Sigma)	CCGGCAACAAGATGAAGAGCACCAACTCGAGTTGGTGCTCTTCATCTTGTTGTTTTT
shMED1-1	RCN0000019799 (TRC1.5 librairy - Sigma)	CCGGGCAGTAAATCAGAAGGTTCATCTCGAGATGAACCTTCTGATTTACTGCTTTTT
shMED1-2	TRCN0000019800 (TRC1.5 librairy - Sigma)	CCGGGCCGAGTTCCTCTTATCCTAACTCGAGTTAGGATAAGAGGAACTCGGCTTTTT
shNIPBL-1	RCN0000146423 (TRC1.5 librairy - Sigma)	CCGGCGGATGCTTGTCTTACAACTACTCGAGTAGTTGTAAGACAAGCATCCGTTTTTTG
shNIPBL-2	TRCN0000148381 (TRC1.5 librairy - Sigma)	CCGGCTCAGGATTCAGACTCCATAACTCGAGTTATGGAGTCTGAATCCTGAGTTTTTTG
shSMC1A-1	TRCN0000062554 (TRC1.5 librairy - Sigma)	CCGGCGACAGATTATCGGACCATTTCTCGAGAAATGGTCCGATAATCTGTCGTTTTTG
shSMC1A-2	TRCN0000062555 (TRC1.5 librairy - Sigma)	CCGGGCCGGGACTGTATTCAGTATACTCGAGTATACTGAATACAGTCCCGGCTTTTTG
shESR1-1	TRCN0000003299 (TRC1.5 librairy - Sigma)	CCGGAGCACCCTGAAGTCTCTGGAACTCGAGTTCCAGAGACTTCAGGGTGCTTTTTT
shESR1-2	TRCN0000003301 (TRC1.5 librairy - Sigma)	CCGGCTCTACTTCATCGCATTCCTTCTCGAGAAGGAATGCGATGAAGTAGAGTTTTT
shHNF4A-1	TRCN0000019189 (TRC1.5 librairy - Sigma)	CCGGCCACATGTACTCCTGCAGATTCTCGAGAATCTGCAGGAGTACATGTGGTTTTT
shHNF4A-2	TRCN0000019190 (TRC1.5 librairy - Sigma)	CCGGGCAGGAACATATGGGAACCAACTCGAGTTGGTTCCCATATGTTCCTGCTTTTT
shFOSL2-1	TRCN0000329767 (TRC1.5 librairy - Sigma)	CCGGCACGGCCCAGTGTGCAAGATTCTCGAGAATCTTGCACACTGGGCCGTGTTTTTG
shFOSL2-2	TRCN0000329710 (TRC1.5 librairy - Sigma)	CCGGGCAGTGAGTATTGGAAGACTTCTCGAGAAGTCTTCCAATACTCACTGCTTTTTG
shFOXA1-1	TRCN0000014878 (TRC1.5 librairy - Sigma)	CCGGGCGAAGTTTAATGATCCACAACTCGAGTTGTGGATCATTAAACTTCGCTTTTT
shFOXA1-2	TRCN0000014879 (TRC1.5 librairy - Sigma)	CCGGGCGTACTACCAAGGTGTGTATCTCGAGATACACACCTTGGTAGTACGCTTTTT
shFOXA2-1	TRCN0000014915 (TRC1.5 librairy - Sigma)	CCGGGAACGGCATGAACACGTACATCTCGAGATGTACGTGTTCATGCCGTTCTTTTT
shFOXA2-2	TRCN0000014916 (TRC1.5 librairy - Sigma)	CCGGCTACGCCAACATGAACTCCATCTCGAGATGGAGTTCATGTTGGCGTAGTTTTT
